# Characterizing niche differentiation among marine consumers with amino acid δ^13^C fingerprinting

**DOI:** 10.1002/ece3.6502

**Published:** 2020-06-30

**Authors:** Thomas Larsen, Thomas Hansen, Jan Dierking

**Affiliations:** ^1^ Max Planck Institute for the Science of Human History Jena Germany; ^2^ GEOMAR Helmholtz Centre for Ocean Research Kiel Kiel Germany

**Keywords:** Baltic Sea, carbon stable isotopes, diet partitioning, fish diets, food web reconstruction, migration tracking, phytoplankton, predator–prey dynamics

## Abstract

Marine food webs are highly compartmentalized, and characterizing the trophic niches among consumers is important for predicting how impact from human activities affects the structuring and functioning of marine food webs. Biomarkers such as bulk stable isotopes have proven to be powerful tools to elucidate trophic niches, but they may lack in resolution, particularly when spatiotemporal variability in a system is high. To close this gap, we investigated whether carbon isotope (δ^13^C) patterns of essential amino acids (EAAs), also termed δ^13^C_AA_ fingerprints, can characterize niche differentiation in a highly dynamic marine system. Specifically, we tested the ability of δ^13^C_AA_ fingerprints to differentiate trophic niches among six functional groups and ten individual species in the Baltic Sea. We also tested whether fingerprints of the common zooplanktivorous fishes, herring and sprat, differ among four Baltic Sea regions with different biochemical conditions and phytoplankton assemblages. Additionally, we investigated how these results compared to bulk C and N isotope data for the same sample set. We found significantly different δ^13^C_AA_ fingerprints among all six functional groups. Species differentiation was in comparison less distinct, due to partial convergence of the species' fingerprints within functional groups. Herring and sprat displayed region‐specific δ^13^C_AA_ fingerprints indicating that this approach could be used as a migratory marker. Niche metrics analyses showed that bulk isotope data had a lower power to differentiate between trophic niches than δ^13^C_AA_ fingerprinting. We conclude that δ^13^C_AA_ fingerprinting has a strong potential to advance our understanding of ecological niches, and trophic linkages from producers to higher trophic levels in dynamic marine systems. Given how management practices of marine resources and habitats are reshaping the structure and function of marine food webs, implementing new and powerful tracer methods are urgently needed to improve the knowledge base for policy makers.

## Graphical abstract

Our study uses stable isotope fingerprinting of amino acid to characterize resource utilization and niche segregation among consumers in the Baltic Sea. We demonstrate that the isotope fingerprinting method (a) can resolve dietary niches at a much higher resolution than bulk stable isotope analysis (b). This methodological advance can improve our understanding of how management practices affect marine ecosystem functioning.

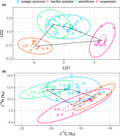

## 1 INTRODUCTION

Direct pressures on marine systems such as increasing temperatures, eutrophication, introduction of nonindigenous species, and overfishing are affecting the performance of individual species and the structure of entire systems. Examples of these consequences include the malnutrition of ecologically and commercially important fish species (Eero et al., 2015), niche shifts following the introduction of nonindigenous species (Ojaveer et al., 2017), and evidence for system‐wide shifts in many regions (Alheit et al., 2005). In this context, identifying organic matter sources at the base of the food web is key for understanding resource partitioning and trophic niche differentiation across time and space.

Resource partitioning among marine species and trophic groups is often poorly understood due to the complexity of marine food webs (Lynam et al., 2017) and methodological constraints (Nielsen, Clare, Hayden, Brett, & Kratina, 2018). Diet identification has traditionally relied on visual taxonomic assessment of stomach and fecal contents (Hyslop, 1980), but visual assessments are now increasingly complemented with DNA metabarcoding (Bowser, Diamond, & Addison, 2013). While the taxonomic resolution of these methods can be high, they only provide instant snapshots of ingested diets provided that the identifiable fragments or DNA sequences are intact. Obtaining intact sequences can be logistically challenging when assessing multiple species over space and time. In comparison, it is possible to integrate dietary histories with stable isotope ratios since the diet‐derived building blocks for animal tissues are sourced over time. Stable isotopes of elements can be informative of diet sources because lighter stable isotopes enter reactions and physical processes at faster rates than heavier stable isotopes, resulting in different isotope ratios among different organic pools. The rate by which elements shifts their isotopic ratios during trophic transfer differs greatly: elements such as carbon and sulfur are used as source tracers because they discriminate (Mittermayr, Hansen, & Sommer, 2014) less compared to nitrogen, which is used as a marker of trophic position (Vander Zanden & Rasmussen, 1999). However, isotope ratios of whole tissues (bulk SIA) often lack source specificity because of variable, and at times, unpredictable isotope discriminates processes and isotope baseline values for different systems (Fry, 2006; Post, 2002). To overcome these limitations, ecologists are increasingly using compound‐specific isotope analyses (CSIA), in which stable isotope ratios are determined for individual compounds, as a complementary approach (Whiteman, Elliott Smith, Besser, & Newsome, 2019).

CSIA of protein amino acids has emerged as one of the most promising approaches to trace the origins and fate of food sources (McClelland & Montoya, 2002; O'Brien, Fogel, & Boggs, 2002). Amino acids (AAs) are among the major conduits of organic carbon in food webs and well suited as a source tracer because metazoans cannot synthesize the carbon backbones of about half of the 20 protein AAs de novo. Instead, metazoans depend on essential amino acids (EAAs) from food sources (McMahon, Fogel, Elsdon, & Thorrold, 2010) or more rarely bacterial symbionts (Larsen, Ventura, et al., 2016). EAAs are powerful source tracers because δ^13^C_EAA_ values remain largely conserved through trophic transfer and because the producers of these EAA, algae, bacteria, fungi, and vascular plants each generate unique δ^13^C_EAA_ patterns or fingerprints (Larsen, Taylor, Leigh, & O'Brien, 2009; Larsen et al., 2013; Scott et al., 2006) (see Box 1 for an illustration). Thus, by analyzing δ^13^C_EAA_ ecologists can circumvent the problem of variable and unknown isotopic fractionation during trophic transfer, but the ability of fingerprints to resolve primary production sources is still unclear. Larsen et al. (2013) compared two dozen species of laboratory cultures comprising of diatoms, cyanobacteria, chrysophytes, chlorophytes, and haptophytes to macroalgae, seagrass, fungi, bacteria, and terrestrial vascular plants and found that of all these groups, phytoplankton displayed the largest intragroup variability in δ^13^C_EAA_ patterns across species and taxonomic groups. Despite some unresolved questions for applying δ^13^C_EAA_ fingerprints in marine environments, they have been applied successfully to track habitat use of fishes with distinct ontogenetic migration patterns (Vane, Larsen, Scholz‐Böttcher, Kopke, & Ekau, 2018), resource and habitat use in marine systems (McMahon, Berumen, & Thorrold, 2012), and proportional contributions of primary production sources to marine consumers (Elliott Smith, Harrod, & Newsome, 2018; Rowe et al., 2019; Vokhshoori, Larsen, & McCarthy, 2014). A recent study on mesozooplankton in the Baltic Sea showed promise in distinguishing between interannual algal assemblages (Eglite et al., 2019). Taken together, these results indicate that δ^13^C_EAA_ fingerprints may be able to provide detailed insights into ecological niches of consumers to a much larger extend than previously realized.

Box 1 **Carbon isotope fingerprinting of essential amino acids (EAAs)**

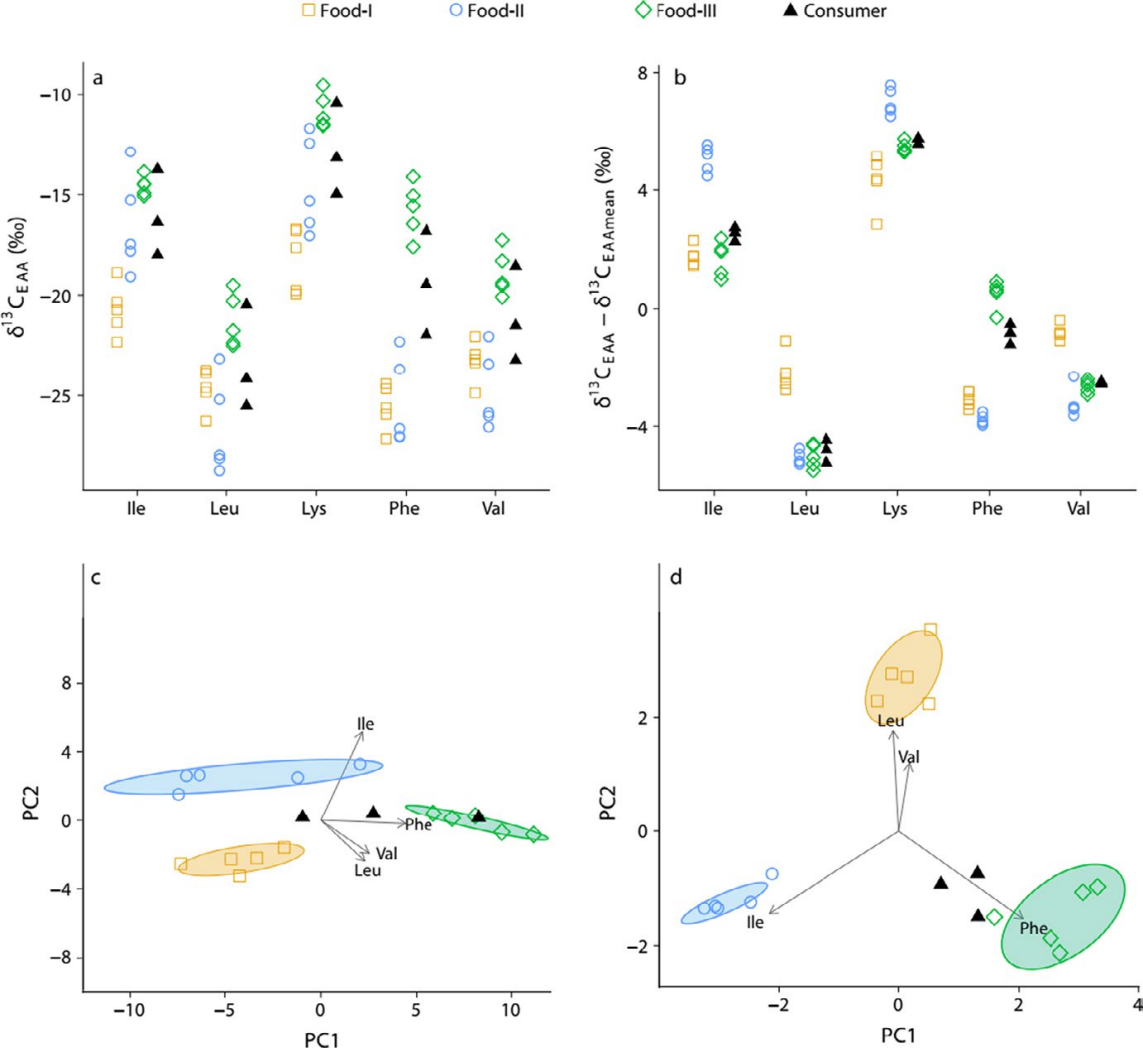

This conceptional model depicts δ^13^C_EAA_ values of consumers feeding in both estuarine and marine habitats. The consumers and their potential food sources mirror δ^13^C baseline values along this salinity gradient, and the δ^3^C_EAA_ intramolecular variability is from Larsen et al. (2015). The two plots in the left pane (a and c) are based on baseline δ^13^C_EAA_ values, and the two plots in the right pane (b and d) are based on δ^13^C_EAA_ values centered to the δ^13^C mean across all EAAs of a given sample. (a) Varying biogeochemical conditions across the estuarine‐marine gradient cause highly variable δ^13^C_EAA_ values. (b) This variability is greatly reduced within each food source when centring the δ^13^C_EAA_ values of each sample to the mean of all five EAAs. (c) To find out which combination of variables explain most of the variability among the three food sources, we applied principal component analysis (PCA), an unsupervised dimensionality reduction method. Prior to the PCA, we omitted lysine because it is the least informative EAA for separating the three food groups. Since the PCA is based on baseline δ^13^C values, the PCA factor scores (PC1 and PC2 coordinates) are influenced by both baseline and intermolecular δ^13^C variability. (d) By using mean‐centered data in the PCA, we have generated a δ^13^C_EAA_ fingerprint where the resulting factor score variability within each group is reduced substantially. By factoring out δ^13^C baseline variability and instead using the source diagnostic power of δ^13^C_EAA_ fingerprinting, it is now evident that regardless of habitat use all three consumers derive most of their dietary EAAs from Food‐III. Abbreviations used on the *x*‐axes in a and b: Ile = isoleucine, Leu = leucine, Lys = lysine, Phe = phenylalanine, and Val = valine.

Exploring further use of CSIA to elucidate changes in basal resources and ecological niches is particularly pertinent for regional seas because of their rapidly warming sea surface temperatures and increasing stressors from anthropogenic activities such as eutrophication and overfishing, with corresponding changes in food webs (Reusch et al., 2018). In this study, we selected the western and central Baltic Sea as a study area because it is a brackish inland sea characterized by strong spatial differences in phytoplankton composition (Eglite et al., 2019; Gasiūnaitė et al., 2005; Wasmund, Dutz, Pollehne, Siegel, & Zettler, 2017) driven by a gradient in hydrographic‐hydrochemical conditions (Naumann et al., 2017). In this sea, food web‐related processes have been identified as driver of changes in ecosystem composition (Möllmann et al., 2009) and declines of key commercial species (Casini et al., 2016; Reusch et al., 2018). Compared to euhaline systems, this brackish water system is characterized by a relatively low diversity (Ojaveer et al., 2010) and a tight coupling of benthic and pelagic food webs (Griffiths et al., 2017; Kiljunen et al., 2020). Across the gradient, the small pelagic fish species herring (*Clupea harengus*) and sprat (*Sprattus sprattus*) are the dominant zooplanktivores, and of large commercial value (Ojaveer, Lankov, Raid, Põllumäe, & Klais, 2018). As zooplanktivores, these species are also natural integrators of pelagic planktonic production.

**FIGURE 1:**
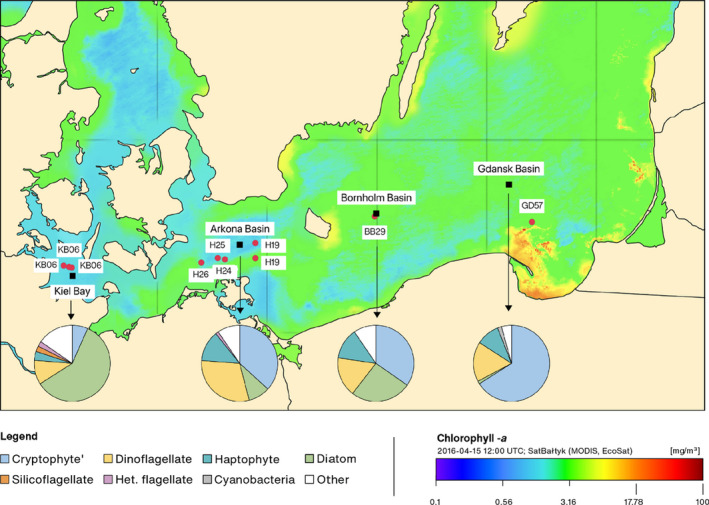
Sampling stations in the Baltic Sea for the AL476 cruise (fauna; filled red circles) and black filled squares for the IOW stations (phytoplankton monitoring; Wasmund et al., 2017). The color gradient on the map shows showing surface concentration of the chlorophyll‐a on 15 April 2016 observed by satellite and supplemented by the results of the ecohydrodynamic model EcoSat (http://satbaltyk.iopan.gda.pl ). The four pie charts present the relative biomass fraction of major taxonomic algal groups integrating monitoring results from three cruises from January to May 2016 (Wasmund et al., 2017). “Het.” is an abbreviation for heterotrophic

To test the power of CSIA to identify niche differentiation among marine consumers in the spatially variable Baltic Sea, we obtained δ^13^C_AA_ values for 10 species. These species encompass both fishes and invertebrates across six different functional groups: suspension feeders, planktivores, benthic predators, benthic flatfishes, and scavengers. Furthermore, to assess the power of the method to identify differences across larger spatial scales, we obtained δ^13^C_AA_ values for herring and sprat from four locations along the Baltic Sea gradient (Figure 1). We first assessed the power of δ^13^C_EAA_ fingerprints to identify (a) trophic niche differentiation among functional groups and among species, and (b) the presence of spatial patterns among planktivorous fishes, positing that different δ^13^C_EAA_ profiles of phytoplankton assemblages would propagate via mesozooplankton to zooplanktivore fishes. Finally, to assess the relative performance of CSIA versus bulk SIA in differentiating functional groups, we obtained bulk isotope (δ^13^C and δ^15^N) values for a subset of the samples assessed with CSIA and compared the niche separation among functional groups with niche metrics analysis (Jackson, Inger, Parnell, & Bearhop, 2011).

## 2 MATERIAL AND METHODS

### 2.1 Study system

The Baltic Sea is a shallow (mean depth 58 m) temperate regional sea, which displays a strong salinity gradient from marine salinity (30 g/kg) at the connection to the North Sea in the west to near freshwater (2 g/kg) in the northeastern inner part (Meier, 2007). The Baltic environmental situation entails strong fluctuations in temperature and light availability, a horizontal salinity gradient and strong vertical stratification, low oxygen conditions in the deep parts of the basins (Carstensen, Andersen, Gustafsson, & Conley, 2014), and an abundant nutrient supply due to eutrophication (Gustafsson et al. 2012), with seasonal minima when nutrients are taken up during phytoplankton blooms. Due to an accumulation of anthropogenic pressures on a level that is expected for other coastal seas, the system has been coined a “time machine for the future coastal oceans” (Reusch et al., 2018).

### 2.2 Fauna sampling

Sampling for this study took place during research cruise AL476 with research vessel ALKOR in April 2016 (see sampling stations in Figure 1). All specimens were measured (total length or diameter to the nearest mm, mass to the nearest g), and ca. 0.5 cm^3^ of muscle tissue was taken for isotope analysis and immediately frozen at −20°C on board of the vessel for further analyses. Our sampling was designed with our two main research questions in mind: (a) can δ^13^C_AA_ fingerprints differentiate feeding niches at functional group and species levels, and (b) is it possible to obtain geographically distinct fingerprints for widely distributed zooplanktivorous fishes. For the first research question, we collected the following species in the two westernmost sites, Kiel Bay and the Arkona Basin: planktivores (herring: *C. harengus* Linnaeus 1758) and (European sprat: *S. sprattus* Linnaeus 1758), pelagic piscivore (Whiting: *Merlangius merlangus* Linnaeus 1758), suspension feeders (Ocean quahog: *Arctica islandica* Linnaeus 1767, Blue mussels: *Mytilus edulis* Linnaeus 1758), benthic‐demersal predatory flatfish (Common dab: *Limanda limanda* Linnaeus 1758, European flounder: *Platichthys flesus* Linnaeus 1758, European plaice: *Pleuronectes platessa* Linnaeus 1758), benthic predators (Common starfish: *Asteria rubens* Linnaeus 1758), and scavengers (Red whelk: *Neptunea antiqua* Linnaeus 1758). For the second research question, we also sample herring and sprat in the two easternmost sites, Bornholm Basin and Gdansk Basin. For further sample characteristics, see Table 1 for a summary and Supplementary S1 for detailed information.

**TABLE 1:** Sampling summary for research cruise AL476 with research vessel ALKOR in April 2016

Basin	Species	Func. group	CSIA (*n*)	BSIA (*n*)	Length mean [min‐max] (cm)	Mass mean [min‐max] (g)
Kiel Bight	*Arctica islandica*	Suspension	5	5	3.6 [3.2–4.0]	12.0 [4.1–20.0]
	*Asterias rubens*	Benthic predator	5	5	8.5 [5.5–12.2]	10.0 [2.9–20.0]
	*Clupea harengus*	Planktivore	5	5	14.4 [12.5–16.5]	22.1 [16.2–29.8]
	*Limanda limanda*	Benthic flatfish	5	5	18.2 [14.5–21.5]	69.2 [32.0–114.0]
	*Neptunea antiqua*	Scavenger	3	3	5.1 [4.3–5.8]	11.2 [6.8–16.1]
	*Platichthys flesus*	Benthic flatfish	5	5	27.8 [25.5–30.0]	225.2 [180.0–278.0]
	*Sprattus sprattus*	Planktivore	5	5	9.6 [7.0–11.5]	7.5 [2.5–11.8]
Arkona Basin	*Asterias rubens*	Benthic predator	5	5	6.2 [5.2–7.6]	8.3 [5.3–11.4]
	*Clupea harengus*	Planktivore	5	5	20.0 [12.0–25.0]	63.9 [11.5–108.0]
	*Merlangius merlangus*	Pelagic piscivore	5	0	31.4 [29.0–35.0]	261.6 [202.0–405.0]
	*Mytilus edulis*	Suspension	5	5	4.2 [2.4–5.4]	5.0 [0.7–9.5]
	*Platichthys flesus*	Benthic flatfish	5	5	26.4 [19.0–37.0]	196.6 [78.0–397.0]
	*Pleuronectes platessa*	Benthic flatfish	5	5	31.4 [27.5–45.0]	381.8 [184.0–987.0]
	*Sprattus sprattus*	Planktivore	5	5	12.5 [11.5–13.0]	14.4 [12.0–16.1]
Bornholm Basin	*Clupea harengus*	Planktivore	5	5	16.6 [15.5–17.0]	33.2 [26.0–38.0]
	*Sprattus sprattus*	Planktivore	5	5	11.7 [11.0–12.5]	10.8 [8.2–12.3]
Gdansk Basin	*Clupea harengus*	Planktivore	5	5	20.0 [17.0–22.5]	47.6 [22.0–66.0]
	*Sprattus sprattus*	Planktivore	5	2	10.4 [9.5–11.0]	7.2 [6.5–8.3]

Note

CSIA indicates the number of specimens analyzed for compound‐specific stable isotope analysis and BSIA the number of specimens analyzed for bulk stable isotope analysis.

### 2.3 Phytoplankton assemblages

Information of phytoplankton communities during the study period was obtained from publicly available plankton monitoring data published by Wasmund et al. (2017). The compiled phytoplankton data from January through May show that the phytoplankton spring bloom in 2016 occurred almost simultaneously in the Belt Sea, Arkona Basin, and Bornholm Basin during the first half of March. The bloom was dominated by diatoms in Kiel Bay and increasingly by *Mesodinium rubrum* (a photosynthetic ciliate that relies on chloroplasts derived from its cryptophyte symbiont (Qiu, Huang, & Lin, 2016)) along a western to eastern latitudinal gradient. We compiled the relative abundance of major algal groups based on the 10 most abundant phytoplankton taxa—see pie charts in Figure 1. The most noticeable trends across the latitudinal gradient are the much greater diatom abundance in Kiel Bight than Gdansk Basin, and vice versa for the cryptophyte group. The total plankton production was smaller in the western than eastern sites; Kiel Bay: 488 µg/L, Arkona Basin: 412 µg/L, Bornholm Basin: 702 µg/L, and Gdansk Basin: 796 µg/L (averages from three cruises January–May) (Wasmund et al., 2017).

### 2.4 Stable isotope analysis

Isotope data are expressed in delta (*δ*) notation:

$$\delta^{i}E_{\mathrm{sample}}=\frac{{\left(\frac{i_{E}}{j_{E}}\right)}_{\mathrm{sample}}-{\left(\frac{i_{E}}{j_{E}}\right)}_{\mathrm{ref}}}{{\left(\frac{i_{E}}{j_{E}}\right)}_{\mathrm{Ref}}}$$

For the element *E*, the ratio of heavy (*i*) to light (*j*) isotope is measured in both sample and references (Coplen & Shrestha, 2016). To express the isotopic data as per mil (‰), they are multiplied by 1,000. The isotope ratios are expressed relative to international standards; Vienna Pee Dee Belemnite (VPDB) for carbon and atmospheric air for nitrogen.

All tissue samples for compound‐specific isotope analysis were freeze‐dried and then hydrolyzed in 6 N HCl at 110°C for 20 hr before derivatizing the AAs to *N*‐acetyl methyl esters (NACME, (Corr, Berstan, & Evershed, 2007) following the protocols by (Larsen, Pollierer, et al., 2016; Larsen et al., 2013). The samples were analyzed at the Leibniz Laboratory at Kiel University. The average standard deviation for the samples, across all AAs was 0.3‰ for δ^13^C (3 injections). The average standard deviation for the internal reference standard norleucine (Nle) was 0.3‰ (3 injections) and 0.2‰ for Pro to 0.6‰ for Ala (4–7 injections) for the in‐house AA references. We obtained well‐defined peaks of the following AAs here categorized into NEAAs and EAAs, respectively: NEAAs: alanine (Ala), asparagine/aspartic acid (Asx), glutamine/glutamic acid (Glx), glycine (Gly), proline (Pro), and serine (Ser) and EAAs: histidine (His), isoleucine (Ile), leucine (Leu), lysine (Lys), methionine (Met), phenylalanine (Phe), threonine (Thr), and valine (Val). Despite tyrosine (Tyr) being an NEAA that is synthesized by animals through hydroxylation of the aromatic side chain of phenylalanine, we here treat it as an EAA because it is minimally fractionated during trophic transfer compared to most other NEAAs. Elemental content and bulk isotope values were determined at the Stable Isotope Facility of the Experimental Ecology Group, GEOMAR, Kiel. The standard deviation for measured stable isotope reference standards in the range 5–15 µg N and 10–140 µg C mass range was ±0.2‰ and ±0.15‰, respectively (*n* = 3). Lipids are depleted in ^13^C relative to other major tissue constituents (DeNiro & Epstein, 1978), which can affect bulk SIA comparisons between consumers with different lipid content. We did not perform lipid extraction prior to stable isotope analyses of tissue samples because this can affect *δ* ^15^N values (Svensson, Schouten, Hopmans, Middelburg, & Damste, 2016). Instead, we arithmetically normalized *δ* ^13^C values using the C/N values following (Kiljunen et al., 2006). For detailed CSIA and bulk SIA methods, see the Supplementary Information. See Table S2 for CSIA δ^13^C values and Table S3 for bulk δ^13^C and δ^15^N values.

### 2.5 Statistical analyses

All statistical analyses were performed in R version 3.5.1 (R‐Development‐Core‐Team, 2018). To assess whether the EAAs in consumers originate from bacteria, fungi or marine phytoplankton, we applied linear discriminant function analysis (LDA) (R: *MASS*) using δ^13^C_EAA_ training data from Larsen et al. (2013). To assess the power of differentiating among functional groups and among species with δ^13^C_EAA_ data, we applied principal component analysis (PCA, R: *vegan*) using mean‐centered δ^13^C_EAA_ values to factor out baseline isotope variability. The mean‐centered value for a given sample was calculated by subtracting the respective mean δ^13^C value of all the EAAs from each individual δ^13^C_EAA_ value. Prior to the PCA, we applied LDA to find the most effective set of independent variables, that is, δ^13^C_EAA_, for predicting category membership. With this set of independent variables, we performed covariance matrix PCA that preserves variance as the range and scale of variables are in the same units of measure. Based on the first and second principal component scores, we used two different approaches to visualize predefined groups; 95% prediction ellipses visualize variability relative to the group centroid, and convex hulls visualize the amount of space taken up by a given group. We applied multivariate analysis of variance (MANOVA, R: *MANOVA*) in conjunction with Pillai's trace to test the null hypothesis that groups have a common centroid in a dependent variable vector space. A rejection of this hypothesis entails that the groups have significantly different δ^13^C_EAA_ patterns or fingerprints. The MANOVA tests were performed on groups with ≥5 specimens. To remove the effect of covariate factors when testing for significant differences between group means, we applied multivariate analysis of covariance (MANCOVA, R: *jmv*). All data for multivariate comparisons were first assessed for homogeneity of variance by using Fligner–Killeen tests (R: *fligner.test*) and visually checked for departures from normality on Q–Q plots. To test for species‐specific δ^13^C differences for each EAA for consumers from Kiel Bight and the Arkona Basin, respectively, we used a one‐way ANOVA with Tukey's HSD test (R: *aov; TukeyHSD*). We determined niche width for both the δ^13^C_EAA_ and bulk datasets with ≥10 specimens. The δ^13^C_EAA_ niche widths are based on the first and second linear discriminant values, and the bulk isotope niche widths are based on δ^13^C (lipid corrected) and δ^15^N values. We determined the niche width overlap between pairs of functional groups by calculating the overlap of the 95% prediction ellipses that are based on the groups' maximum‐likelihood estimated mean and covariance matrices (R:SIBER: *RmaxLikOverlap*) (Jackson et al., 2011). We also plotted the 95% confidence interval of the groups' bivariate means, which is commonly referred to as the standard ellipse area (SEA). The niche width distribution of the entire community was defined according to community‐level Layman metrics, that is, the total area of the convex hull encompassing the group means (TA_c_) (Layman, Arrington, Montana, & Post, 2007). To make the community niche space comparable for the two isotope methods, we took the ratio between TA_c_ and the combined SEAs. A higher number signifies a greater niche width separation.

## 3 RESULTS

### 3.1 Biosynthetic origins of the essential amino acids

**FIGURE 2:**
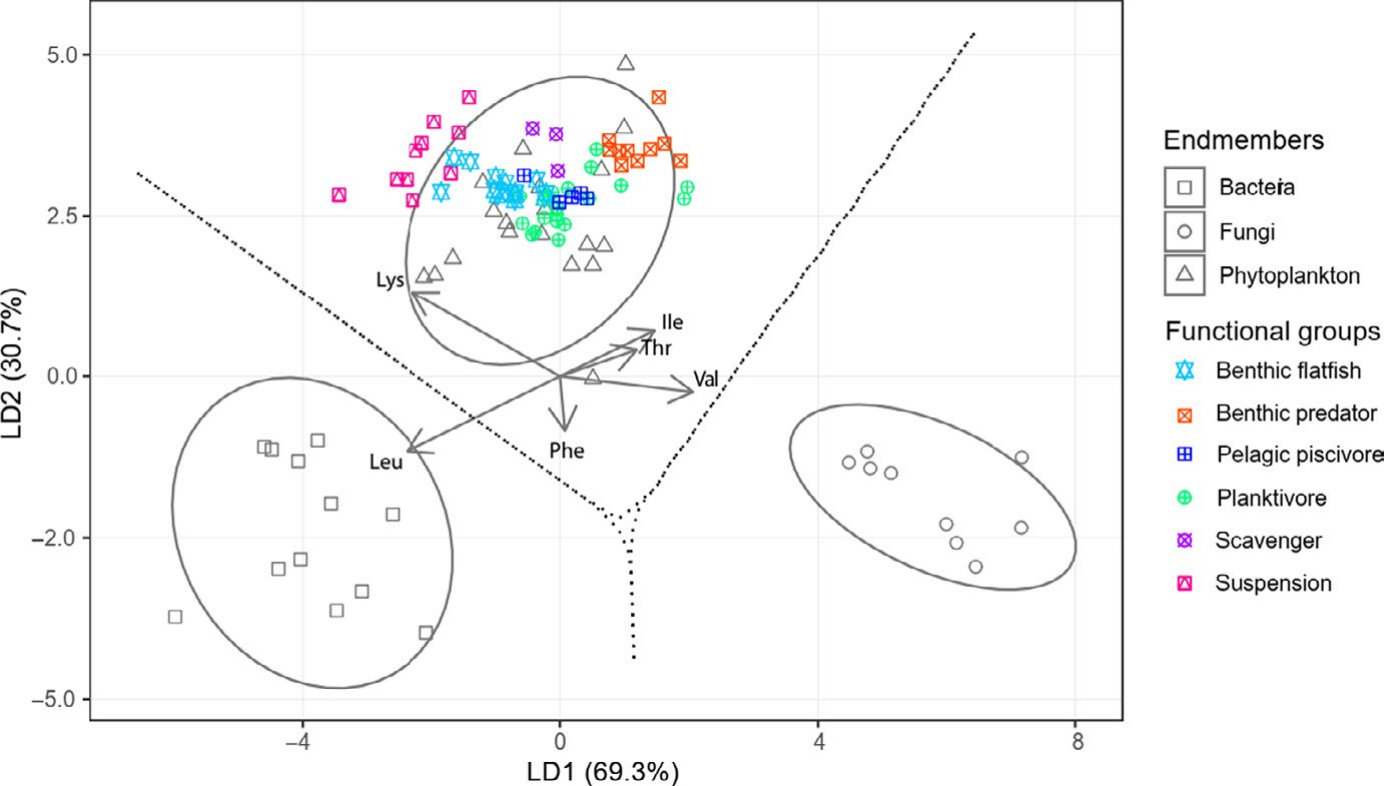
Linear discriminant function analysis based on δ^13^C_EAA_ values of training data comprising of bacteria, fungi and marine phytoplankton (Larsen et al., 2013) and consumers from this study. The phytoplankton comprise of eight diatom samples (D1‐D5; N1‐N3), four chrysophytes (X1‐X4), four haptophytes (H1 – H4), two chlorophytes (K1 & K2), and one cryptophyte (Y1)— see Larsen et al. (2013) for sample codes. The ellipses represent 95% confidence intervals of each endmember, and the arrows represent the relative weightings of the independent variables for creating the discriminant function. Amino acid abbreviations: isoleucine (Ile), leucine (Leu), lysine (Lys), phenylalanine (Phe), threonine (Thr), and valine (Val)

According to our LDA using training data of broad phylogenetic groups, phytoplankton were the primary EAA source for all consumers in Kiel Bay and Arkona Basin; contributions from bacteria and fungi were small or possibly absent (Figure 2). The discrete clustering of most functional groups indicates that they were supported by different phytoplankton sources at the base of the food web, here listed in terms of association along the along the first linear discriminant: suspension feeders, benthic flatfishes, scavengers, pelagic piscivores, planktivores, and benthic predators.

### 3.2 **Differentiating functional groups and species with** δ^13^C**_EAA_ fingerprinting**

**FIGURE 3:**
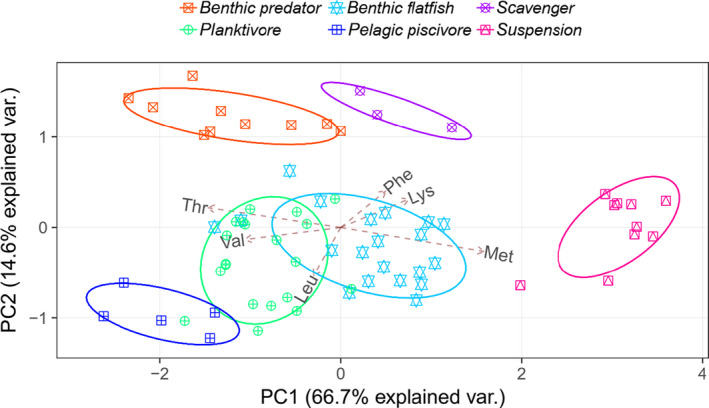
Principal component analysis for functional groups using mean‐centered δ^13^C_EAA_ values of consumers from Kiel Bay and Arkona Basin. Values in parentheses are the percentage variations accounted by each axis. The two axes account for 82% of the variations. The ellipses signify 95% confidence boundaries for each group. Amino acid abbreviations: leucine (Leu), lysine (Lys), methionine (Met), phenylalanine (Phe), threonine (Thr), and valine (Val)

**FIGURE 4:**
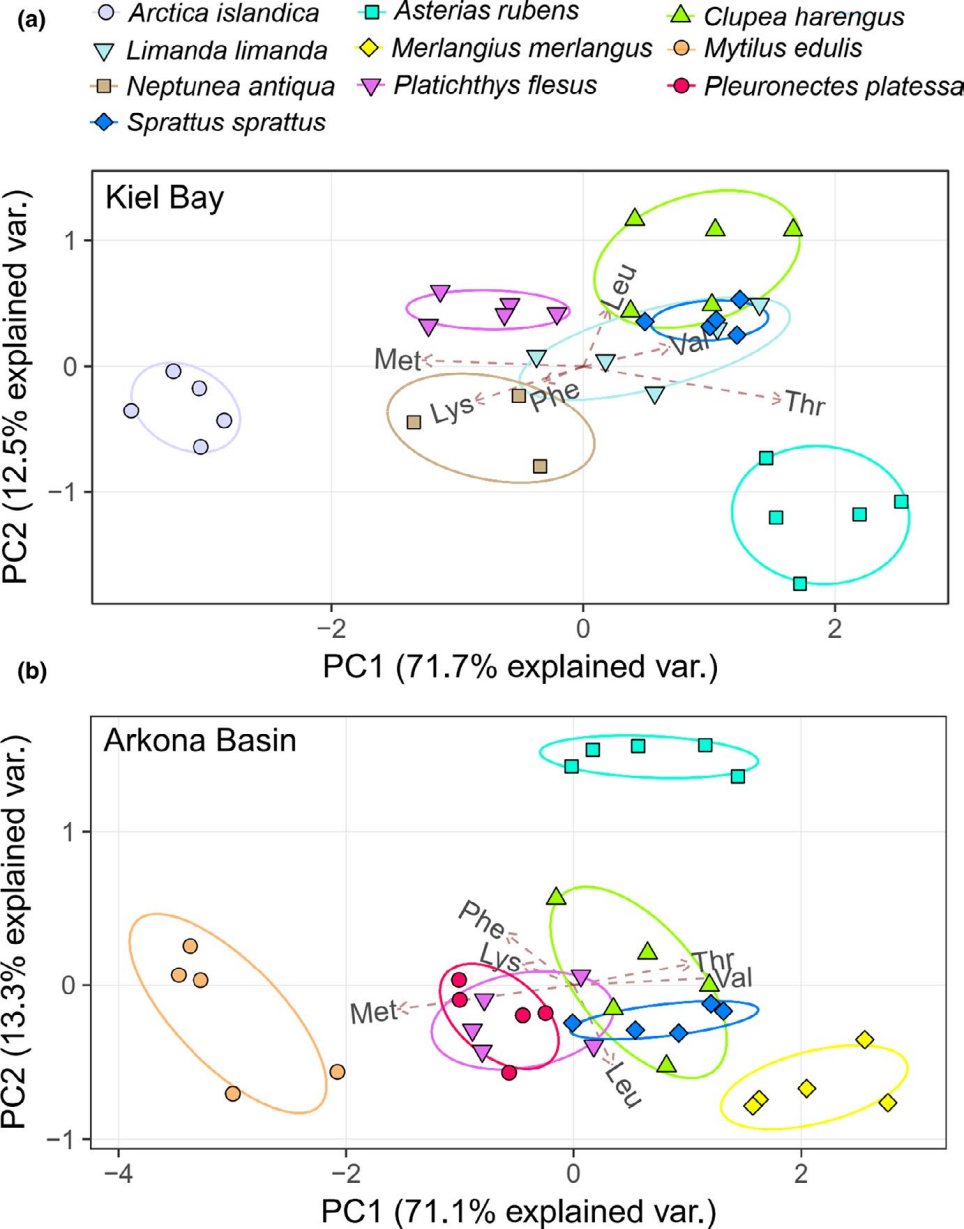
Principal component analysis for species using δ^13^C_EAA_ values centered to the EAA mean of consumers from Kiel Bay (a) and Arkona Basin (b), respectively. Values in parentheses are the percentage variations accounted by each axis. In (a and b), the first two axes account for 84% and 83% of the variations, respectively. The ellipses signify 95% confidence boundaries for each group. Amino acid abbreviations: isoleucine (Ile), leucine (Leu), lysine (Lys), methionine (Met), phenylalanine (Phe), threonine (Thr), and valine (Val)

For both the Kiel Bay and Arkona Basin datasets, all six functional groups cluster separately based on their δ^13^C_EAA_ fingerprints (Figure 3). Suspension feeders belong to the most distinct and isolated group; scavengers and benthic predators cluster adjacent to one another; pelagic piscivores, planktivores, and benthic flatfishes also cluster adjacently. The median values of the five largest groups were significantly different (Pillai's Trace = 1.55, *F*_6,112_ = 63.6; *p* < .001). Our comparison between species for each site shows that most sprat and herring specimens have similar principal component scores for Kiel Bay (Figure 4a) and Arkona Basin (Figure 4b). Sprat and herring cluster adjacent to the three species of benthic flatfishes. Starfish and the two suspension‐feeding species each group in distinct and isolated clusters. We did not test for differences in median values at a species level because we had five or less specimens of each species. For both sites, the most effective set of variables for predicting species membership are Thr, Val, and Met (Figure 4a,b).

### 3.3 δ^13^C**_EAA_ fingerprints across Baltic regions**

**FIGURE 5:**
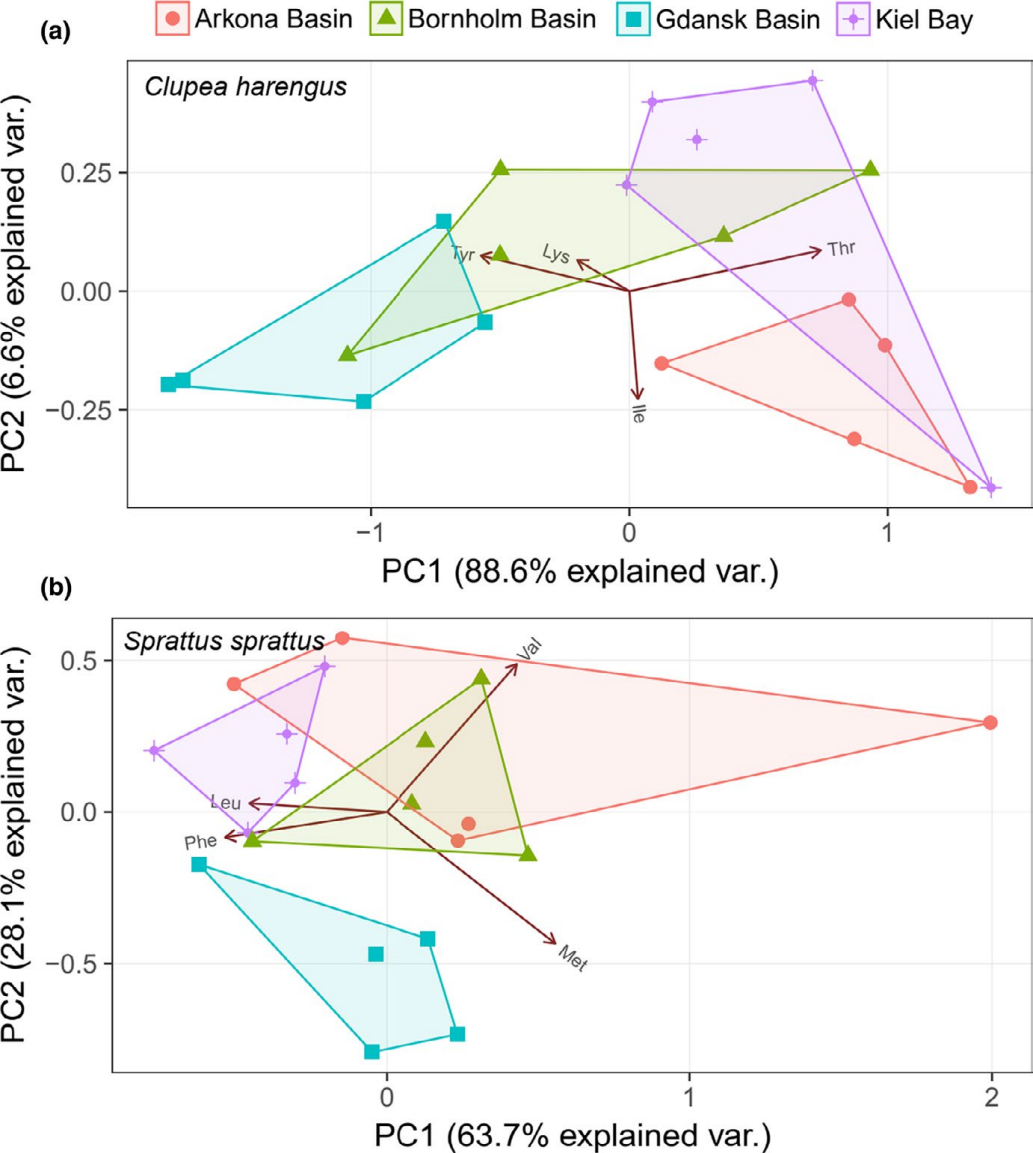
Principal component analysis (PCA) with δ^13^C_EAA_ values centered to the EAA mean of herring (a) and sprat (b), respectively. The convex hulls represent the maximum range in PC1 and PC2 scores for each of the four sampling locations. The most important EAAs for variations among locations are displayed in two first ordination components. Values in parentheses are the percentage variations accounted by each axis. In (a and b), the first two axes account for 95% and 92% of the variations, respectively. For a PCA with both species, see Figure S1. Amino acid abbreviations: isoleucine (Ile), leucine (Leu), lysine (Lys), methionine (Met), phenylalanine (Phe), threonine (Thr), tyrosine (Tyr) and valine (Val)

The δ^13^C_EAA_ fingerprints of clupeids from the four Baltic Sea regions show region‐specific clustering of most herring and sprat specimens (Figure 5a,b). The separation is stronger for herring (Pillai's trace = 1.05, *F*_6,32_ = 5.9; *p* < .001) than for sprat (Pillai's trace = 0.90, *F*_6,32_ = 4.4; *p* < .01) due to larger principal component variability of the Arkona Basin specimens. The region‐specific separation becomes weaker when joining the two clupeid species (see Figure S1). To assess how ontogenetic factors may have influenced the observed δ^13^C_EAA_ fingerprints, we incorporated mass of individual specimens as a covariate using MANCOVA. The significance of these tests is similar to those obtained with MANOVA for both herring (Pillai's Trace = 1.06, *F*_6,30_ = 5.6; *p* < .001) and sprat (Pillai's trace = 0.94, *F*_6,30_ = 4.4; *p* < .01).

### 3.4 Niche differentiation with compound and bulk isotope methods

**FIGURE 6:**
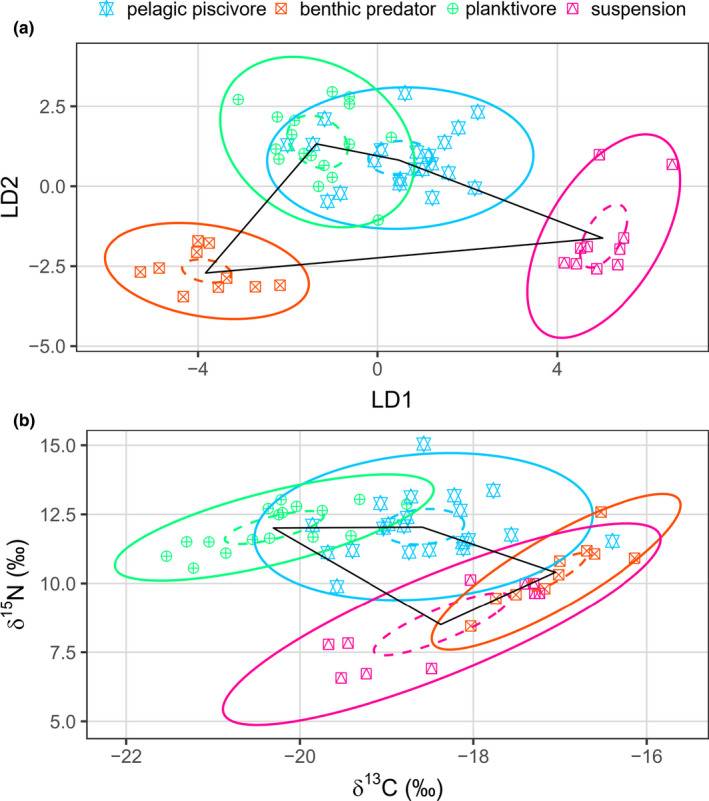
Niche spaces of Kiel Bay and Arkona Basin functional groups based on multivariate δ^13^C_EAA_ (a) and bivariate bulk isotope (b; δ^13^C_lipid corrected_ and δ^15^N) values. To represent the δ^13^C_EAA_ data in a bivariate space (see Figure 3 for the independent variables), we used the first two linear discriminant scores encompassing 97.8% of the variability. The groups depicted here contain ≥10 specimens. The niche spaces are visualized by 95% confidence interval around the bivariate means, also called the standard ellipse area (SEA; inner ellipses with broken lines) and 95% prediction ellipses (outer ellipses with full lines), respectively. The convex hulls encompassing the group means are denoted TA_c_ for the total area of each community. The community niche space is generally more separated for δ^13^C_EAA_ than bulk isotopes; for example, there is only one overlap of the prediction ellipses between functional groups in the δ^13^C_EAA_ biplot, but four overlaps in the bulk biplot

The differentiation of the functional groups' feeding niches, visualized based on biplots of δ^13^C_EAA_ discriminant scores and bulk δ^13^C and δ^15^N isotope data, is shown in Figure 6a,b. The comparison of the groups' niche areas shows that benthic flatfishes and planktivores overlap in both the δ^13^C_EAA_ (38.3%) and bulk (34.6%) biplots. In contrast, while there are no other group overlaps in δ^13^C_EAA_ biplot, there are several group overlaps in the bulk biplot: benthic predators and suspension feeders (22.4%), benthic flatfishes and benthic predators (6.7%), and benthic flatfishes and suspension feeders (9.2%). Moreover, the greater TA_c_ to SEA ratio for δ^13^C_EAA_ (1.68) than bulk isotope (0.72) indicates a higher level of niche separation for the latter than the former.

To investigate niche differentiation, we pooled Kiel Bight and Arkona Basin specimens because on a species level, baseline δ^13^C and δ^15^N differences between the two locations are inconsistent. For example, European flounder is significantly more ^13^C enriched in Kiel Bight than Arkona Basin (*t*_7_ = −4.7, *p* = .0023), but herring is not (*t*_8_ = 1.4, *p* = .20). There are no significant ^15^N differences between the two regions, for example, European flounder (*t*_7_ = −0.43, *p* = .68) and herring (*t*_8_ = 1.5, *p* = .18).

## 4 DISCUSSION

With accelerating global and regional environmental changes, improved understanding of food web structures is essential to project the corresponding changes of biological systems (Barth, Walter, Robbins, & Pasulka, 2020; Kortsch, Primicerio, Fossheim, Dolgov, & Aschan, 2015). Here, we provide a systematic assessment of the potential of CSIA to provide insights into resource partitioning and trophic niche differentiation among marine consumers in the Baltic Sea; a rapidly changing sea with a strong spatial environmental gradient.

### 4.1 Understanding niche differentiation and resource partitioning with CSIA

Our results show that the δ^13^C_EAA_ fingerprinting method holds considerable potential for identifying feeding differences in marine habitats. In our two westernmost Baltic locations, the Kiel Bay and the Arkona Basin, we were able to identify niche differentiation among all putative functional groups, as well as most species. This differentiation is in agreement with previous knowledge based on traditional methods like stomach content analysis, for example, Hislop et al. (1997). Species with similar modes of feeding clustered closely. It is surprising, however, that sea stars clustered very differently than bivalves, considering that blue mussels are considered a major prey (Sommer, Meusel, & Stielau, 1999). We posit that such mismatches do not pertain to limitations of the fingerprinting method, but rather limited sampling and analysis of relevant endmembers because sea stars also feed on other invertebrates such as sponges, snails, and isopods (Anger, Rogal, Schriever, & Valentin, 1977). Similarly, the closer clustering of benthic flatfishes with planktivorous fishes than other benthic piscivores may be related to similar phytoplankton sources fueling their respective compartments of the food web. However, a more systematic sampling approach would be required to more fully characterize this benthic‐pelagic coupling. Taken together, our results highlight the potential of δ^13^C_EAA_ fingerprinting to elucidate the dietary niches of marine consumers, and how fluxes of carbon and nutrients from primary producers to detritus and consumers structure marine ecosystems (Cebrian, 1999; Lartigue & Cebrian, 2012).

The highly dynamic and complex nature of marine food webs can make it challenging to assess trophic relationships between consumers and producers, particularly on a taxon‐specific level (Armengol, Calbet, Franchy, Rodríguez‐Santos, & Hernández‐León, 2019; Woodward, Speirs, Hildrew, & Hal, 2005). The clear spatial and trophic group differences observed in our study underscore the potential of δ^13^C_EAA_ fingerprinting to determine the trophic basis of production, that is, how particular production sources are linked to consumers. At the same time, it is important bearing in mind that consumer fingerprints will lag behind primary producer fingerprints and that lower level consumers will integrate more recent photosynthates in their tissue than higher level consumers. Hence, frequent sampling would be needed to establish a more holistic picture of trophic connectivity and niche differentiation. Likewise, it will be critical for future studies to establish a reference phytoplankton library based on well‐characterized in situ algal assemblages and single‐species cultures. Increased application of this method to identify the taxonomic groups fueling production on higher trophic levels could improve our understanding of trophic links in many marine food webs and reduce the current bias toward larger prominent species feeding on clearly identifiable food items.

The fingerprinting method is well suited for quantifying inconspicuous sources because laboratory cultures of bacteria, phytoplankton, and other potential endmembers can be used as a proxy for in situ samples (Arthur, Kelez, Larsen, Choy, & Popp, 2014; Larsen et al., 2013; Rowe et al., 2019). For example, the fingerprinting method has yielded invaluable insight into detritus‐based energy channels in soil food webs (Larsen, Pollierer, et al., 2016; Pollierer et al., 2019). Our study did not examine detrital feeders, but bacterial and fungal EAA contributions were undetectable even among benthic and suspension feeders. Since dead phytoplankton biomass usually undergoes a distinct succession of biotic activity and chemical decomposition (Azam & Malfatti, 2007; Biddanda, 1988), we attribute the lack of bacterial fingerprints to two independent processes. First, bacteria lack certain sterols and fatty acids essential for most metazoans (Phillips, 1984), which may explain why consumers such as the ocean quahog feed on recent primary production sources rather than organic matter from surface sediments (Erlenkeuser, 1976; Larsen, Yokoyama, & Fernandes, 2018). Second, the rate by which bacteria rework phytoplankton‐derived EAAs appears to be very slow possibly because microbes assimilate them directly into their tissue rather than synthesizing them de novo (Hannides, Popp, Choy, & Drazen, 2013; Larsen et al., 2015). Our results confirm that tracing detrital‐based energy channels in marine food webs is challenging and may require additional tracer techniques such as bacterial fatty acid biomarkers (Hayakawa, Handa, Kawanobe, & Wong, 1996; Taipale et al., 2015) and DNA metabarcoding of gut content (Fernández‐Álvarez, Machordom, García‐Jiménez, Salinas‐Zavala, & Villanueva, 2018). While the latter two methods are unsuited for quantifying relative nutritional contributions, they provide important information on how detrital processes enter and alter marine energy channels.

### 4.2 Assessing spatial differences in marine consumers and food webs with CSIA

Spatial isotope differences of marine consumers can inform about underlying differences in the organic matter at the base of food webs, as well as migration patterns of individuals (Hansson et al., 1997; McMahon et al., 2012; Torniainen et al., 2017). The geographically distinct δ^13^C_EAA_ fingerprints of herring and sprat observed in our study would be consistent with limited mixing among schools from the different locations, that is, spatial population structuring, in combination with the presence of different phytoplankton assemblages or isotopic baselines among locations. This corresponds well with monitoring studies of phytoplankton highlighting the change in assemblages along the environmental gradient in the Baltic Sea, and with different baselines linked to spatially variable terrestrial organic matter inputs (Rolff & Elmgren, 2000; Wasmund et al., 2017) (Figure 2). The additional observation of substantial variability within the same locations for both sprat and herring could be related to size‐related differences in feeding (Casini, Cardinale, & Arrhenius, 2004; Kleppel, 1993; Last, 1989) as well as differences in migrations, both between areas (Aro, 1989; Gröhsler, Oeberst, Schaber, Larson, & Kornilovs, 2013; Jørgensen, Hansen, Bekkevold, Ruzzante, & Loeschcke, 2005) and in the case of herring between coastal and offshore areas during spawning runs (Šaškov, Šiaulys, Bučas, & Daunys, 2014). Our finding suggests that with further development, δ^13^C_EAA_ fingerprinting have the potential to complement telemetric (Chittenden, Ådlandsvik, Pedersen, Righton, & Rikardsen, 2013; Pincock, Welch, McKinley, & Jackson, 2010) and bulk isoscape (Soto, Wassenaar, & Hobson, 2013; St. John Glew, 2019; Torniainen et al., 2014, 2017) approaches to track migration of single species in offshore systems, as well as novel migration trackers such as δ^15^N_ AA_ (Matsubayashi et al., 2020) and bulk radiocarbon analysis (Larsen et al., 2018). It could also provide further and much needed insight into dietary response to changing physiochemical conditions (Casini et al., 2004; Kulke, 2018),

When we pooled the two clupeid species, the spatial differentiation of the δ^13^C_EAA_ fingerprints weakened considerably. Although the two clupeid species have substantial dietary overlap as corroborated by our results (Figures 3 and 4), it is important to note that both species differ in their dietary preferences. For example, adult herring are not strictly zooplanktivorous; they can opportunistically shift from pelagic to benthic prey by feeding on nektobenthos, that is, consumers such as mysids and amphipods that tend to migrate daily in the water column (Casini, Bartolino, Molinero, & Kornilovs, 2010; Kiljunen et al., 2020). In contrast, all size classes of sprat are strictly zooplanktivorous (Casini et al., 2004). By pooling the two species, we therefore increased variability within each locations. The resulting loss in spatial differentiation is in line with a previous δ^13^C_EAA_ fingerprinting study in the southern Baltic Sea that found poor spatial differentiation after pooling multiple zooplankton species with different dietary preferences (Eglite et al., 2019). These results underline that to leverage the full power of the fingerprinting approach to track migratory patterns, it is important to focus on single species.

### 4.3 Dietary niche differentiation with compound‐specific and bulk isotope approaches

Our ability to answer research questions in trophic ecology and food web studies depends on methodological approaches (Nielsen et al., 2018). EAAs are among the most powerful carbon tracers because EAA carbon backbones are usually passed through multiple trophic levels with minimal modifications in contrast to bulk carbon. The substantially higher differentiation among functional groups and species with the δ^13^C_EAA_ than the bulk isotope approach confirms the usefulness of EAA as high fidelity source tracers. At the same time, it is important to bear in mind that the EAA and bulk isotope approaches are not directly comparable. As demonstrated by our results, the multidimensional δ^13^C_EAA_ niche space is powerful to delineate among primary producers at the base of the food chain, consumers supported by these different sources, and spatial differences among the same organisms from areas with different baselines. This advantage is highlighted by the differentiation between diet sources that are nearly indistinguishable in terms of δ^13^C baselines in past studies, such as marine phytoplankton and kelp (Vokhshoori et al., 2014). In comparison, the isotopic niches identified with the bulk method were less distinguishable, but may hold more easily interpretable information regarding trophic position and terrestrial versus marine or benthic versus pelagic production. Moreover, the lower costs per analyzed sample and the larger repository of reference data (e.g. Pethybridge et al., 2018; de la Vega, Jeffreys, Tuerena, Ganeshram, & Mahaffey, 2019) compared to EAA can be practical considerations in particular for temporal comparisons or studies requiring large sample numbers (e.g., high spatial‐temporal resolution). Ultimately, whether EAA or bulk SIA is the best approach will therefore strongly depend on the study question at hand; complementary use of both methods may in many cases be the optimum solution.

### 4.4 Perspectives

Our study highlights the applicability of δ^13^C_EAA_ fingerprinting in a regional sea with strong salinity and temperature gradients by differentiating among the trophic niches of both functional groups and species at an unprecedented resolution, and by identifying spatial fingerprinting differences of widely distributed species. These differences are likely driven by regional differences in basal resources, that is, algal composition, and the strength of trophic links between various phytoplankton producers and consumers. Our study also highlights how CSIA can provide new insights into food web structuring in spatially and temporally dynamic systems, and thus complement traditional tools in trophic ecology, including insights that are complementary to those from the “traditional” bulk stable isotope analysis.

Current marine food webs are predicted to be fragile and susceptible to structural changes with consequent alterations in the functioning of the ecosystem (Marina et al., 2018). As environmental changes are accelerating, it is crucial to understand whether and how quickly marine food webs can adapt to changes in phytoplankton assemblages (Barth et al., 2020) and top predator abundances (Kortsch et al., 2015). For this reason, it is key identifying and quantifying feeding interactions across trophic levels, from phytoplankton to zooplankton to higher trophic levels, but many of these interactions remain crucial knowledge gaps (Griffiths et al., 2017). The combination of δ^13^C_EAA_ and the more affordable bulk stable isotope analysis holds considerable promise to address these gaps in the future.

## CONFLICT OF INTEREST

None declared.

## AUTHOR CONTRIBUTIONS

**Thomas Larsen:** Conceptualization (equal); data curation (lead); formal analysis (lead); funding acquisition (supporting); investigation (equal); methodology (lead); project administration (supporting); visualization (lead); writing – original draft (lead). **Thomas Hansen:** Formal analysis (supporting); funding acquisition (supporting); investigation (supporting); methodology (supporting); writing – original draft (supporting). **Jan Dierking:** Conceptualization (equal); data curation (supporting); formal analysis (supporting); funding acquisition (lead); investigation (equal); methodology (supporting); project administration (lead); visualization (supporting); writing – original draft (supporting).

## Supporting information

Supplementary Material

## Data Availability

Data associated with this paper are available in the Supplementary Information and DRYAD: https://doi.org/10.5061/dryad.crjdfn321.
