# Supplementary material for: Characterizing niche differentiation among marine consumers with amino acid δ13C fingerprinting
Source: Ecol Evol. 2020 Jun 30;10(14):7768–82. doi: 10.1002/ece3.6502 (PMC7391304; doi:10.1002/ece3.6502)

**Supplementary Material and Methods**

**Compound specific isotope analysis.** To account for carbon added during derivatization and variability of isotope fractionation during analysis, we also derivatized and analysed a mixture of pure amino acids (AAs) with known δ^13^C values. The accuracy and precision of the instrument were also determined daily by measuring this AA reference mixture between samples. Nor-leucine (Nle) was added to each sample before derivation as an internal standard. The samples and reference mixture were stored up to 2 months at -20ºC before isotope analysis. At Leibniz Laboratory at Kiel University, the AA derivatives were injected with an autosampler into an Agilent Single Taper Ultra Inert Liner set at 280ºC and separated on a Varian VF-35ms GC column (30 m length, 0.32 mm inner diameter and 1.00 µm film thickness) installed on an Agilent 6890N gas chromatograph (GC). GC conditions were set as follows to optimize peak separation and shape: initial temperature at 80°C held for 1 min; ramped to 135°C at 20°C min^-1^; ramped to 160°C at 5°C min^-1^, held for 3min; then ramped to 300°C at 8°C min^-1^ and held for additional 3 min. The separated AA peaks were combusted in a GC-III combustion (C) interface, and measured as CO_2_ with a MAT 253 (Thermo-Finnigan Corporation) isotope ratio mass spectrometer (IRMS). Calibration of our internal standard AA-mixture was carried out against the known *δ*^13^C values of A4 mixture (available from A. Schimmelmann, Biogeochemical Laboratories, Indiana University, USA). In regard to analytical uncertainty, the average reproducibility for the internal reference standard nor-leucine (Nle) was 0.3‰ (n = 3 for each batch) and the in-house AA standards ranged from 0.2‰ for Pro to 0.6‰ for Ala (n = 4–7 for each batch). We obtained well defined peaks of the following AAs here categorized into NEAA and EAA, respectively. NEAA: alanine (Ala), asparagine/aspartic acid (Asx), glutamine/glutamic acid (Glx), glycine (Gly), proline (Pro), and serine (Ser). EAA histidine (His), isoleucine (Ile), leucine (Leu), lysine (Lys), methionine (Met), phenylalanine (Phe), threonine (Thr), and valine (Val). Despite tyrosine (Tyr) being an NEAA that is synthesized by animals through hydroxylation of the aromatic sidechain of phenylalanine, we here treat it as an EAA because it fractionates very little during trophic transfer.

**Bulk isotope analysis.** Approximately 60-100 µg dry mass of each sample was weighed into small tin capsules (3.2x4.0 mm, Hekatech, Germany). Samples were analysed by a customized, high sensitivity elemental analyser connected to an stable isotope ratio mass spectrometer (DeltaPlus Advantage, Thermo Fisher Scientific, Germany) as described by Hansen and Sommer (2007). System calibration was implemented by the combustion of International Atomic Energy Agency (IAEA-N1, IAEA-N2, IAEA-N3 for *δ*^15^N) and National Institute of Standards and Technology (NBS-22 and NBS-600 for *δ*^13^C) compounds. Acetanilide p.a. (Merck, Germany) was used as an internal standard after every sixth sample within each sample run. The overall standard deviation (SD) for the low measurement range 2.5-8.0 µg N and 5.0-80 µg C was ±0.25 ‰ and ±0.2‰, respectively. The overall SD for the higher measurement range 5.0-15.0 µg N and 10.0-140 µg C was ±0.2 ‰ and ±0.15‰, respectively. We did not perform lipid extraction prior to stable isotope analyses of tissue samples because this can affect *δ*^15^N values (Svensson *et al.* 2016). Instead, we applied lipid correction to *δ*^13^C values with C/N values larger than 3.3 (indicating elevated lipid content) following Post *et al.* (2007). See Supplementary Table S3 for bulk δ^13^C and δ^15^N values.

References

Hansen, T. & Sommer, U. (2007) Increasing the sensitivity of δ^13^C and δ^15^N abundance measurements by a high sensitivity elemental analyzer connected to an isotope ratio mass spectrometer. *Rapid Communications in Mass Spectrometry,* **21,** 314-318.

Post, D.M., Layman, C.A., Arrington, D.A., Takimoto, G., Quattrochi, J. & Montaña, C.G. (2007) Getting to the fat of the matter: models, methods and assumptions for dealing with lipids in stable isotope analyses. *Oecologia,* **152,** 179-189.

Svensson, E., Schouten, S., Hopmans, E.C., Middelburg, J.J. & Damste, J.S.S. (2016) Factors controlling the stable nitrogen isotopic composition (δ^15^N) of lipids in marine animals. *PLoS ONE,* **11**.

**Supplementary Tables**

Table S1. Species description of samples collected during the AL476 in April 2016.

| **Identifier** | **Sampling date** | **Station** | **Species** | **TL (cm)** | **Mass (g)** |
| --- | --- | --- | --- | --- | --- |
| CD1K | 13/04/2016 | KB06 | *Limanda limanda* | 14.5 | 32 |
| CD3K | 13/04/2016 | KB06 | *Limanda limanda* | 17.0 | 54 |
| CD4K | 13/04/2016 | KB06 | *Limanda limanda* | 18.5 | 66 |
| CD6K | 13/04/2016 | KB06 | *Limanda limanda* | 19.5 | 80 |
| CD8K | 13/04/2016 | KB06 | *Limanda limanda* | 21.5 | 114 |
| CD1F | 13/04/2016 | KB06 | *Platichthys flesus* | 28.0 | 236 |
| CD2F | 13/04/2016 | KB06 | *Platichthys flesus* | 30.0 | 278 |
| CD1M | 13/04/2016 | KB06 | *Arctica islandica* | 3.99 | 20 |
| CD3M | 13/04/2016 | KB06 | *Arctica islandica* | 3.17 | 4.09 |
| CD4M | 13/04/2016 | KB06 | *Arctica islandica* | 3.34 | 8.39 |
| CD5M | 13/04/2016 | KB06 | *Arctica islandica* | 3.66 | 12.52 |
| CD6M | 13/04/2016 | KB06 | *Arctica islandica* | 3.78 | 15.15 |
| CD1St | 13/04/2016 | KB06 | *Asterias rubens* | 12.36 | 20 |
| CD2St | 13/04/2016 | KB06 | *Asterias rubens* | 5.46 | 2.85 |
| CD4St | 13/04/2016 | KB06 | *Asterias rubens* | 8.35 | 8.77 |
| CD7St | 13/04/2016 | KB06 | *Asterias rubens* | 9.44 | 12.53 |
| CD9St | 13/04/2016 | KB06 | *Asterias rubens* | 7.12 | 5.77 |
| CD1Sn | 13/04/2016 | KB06 | *Neptunea antiqua* | 5.12 | 10.65 |
| CD2Sn | 13/04/2016 | KB06 | *Neptunea antiqua* | 5.78 | 16.1 |
| CD3Sn | 13/04/2016 | KB06 | *Neptunea antiqua* | 4.31 | 6.8 |
| CD5F | 13/04/2016 | KB06 | *Platichthys flesus* | 28.5 | 242 |
| CD6F | 13/04/2016 | KB06 | *Platichthys flesus* | 27.0 | 190 |
| CD8F | 13/04/2016 | KB06 | *Platichthys flesus* | 25.5 | 180 |
| CD14H | 13/04/2016 | KB06 | *Clupea harengus* | 13 | 16.18 |
| CD1H | 13/04/2016 | KB06 | *Clupea harengus* | 16.5 | 28.44 |
| CD5H | 13/04/2016 | KB06 | *Clupea harengus* | 16 | 29.77 |
| CD7H | 13/04/2016 | KB06 | *Clupea harengus* | 14 | 19.34 |
| CD8H | 13/04/2016 | KB06 | *Clupea harengus* | 12.5 | 16.61 |
| CD163S | 13/04/2016 | KB06 | *Sprattus sprattus* | 8 | 3.77 |
| CD168S | 13/04/2016 | KB06 | *Sprattus sprattus* | 11.5 | 11.78 |
| CD172S | 13/04/2016 | KB06 | *Sprattus sprattus* | 10.5 | 8.56 |
| CD174S | 13/04/2016 | KB06 | *Sprattus sprattus* | 11 | 10.98 |
| CD175S | 13/04/2016 | KB06 | *Sprattus sprattus* | 7 | 2.51 |
| CD11M | 14/04/2016 | H26 | *Mytilus edulis* | 3.89 | 3.59 |
| CD13M | 14/04/2016 | H26 | *Mytilus edulis* | 4.28 | 4.03 |
| CD14M | 14/04/2016 | H26 | *Mytilus edulis* | 2.39 | 0.74 |
| CD7M | 14/04/2016 | H26 | *Mytilus edulis* | 5.37 | 9.51 |
| CD8M | 14/04/2016 | H26 | *Mytilus edulis* | 5.01 | 7.03 |
| CD11F | 14/04/2016 | H26 | *Platichthys flesus* | 19 | 78 |
| CD10So | 14/04/2016 | H26 | *Pleuronectes platessa* | 28.5 | 258 |
| CD11So | 14/04/2016 | H26 | *Pleuronectes platessa* | 28 | 216 |
| CD12So | 14/04/2016 | H26 | *Pleuronectes platessa* | 28 | 264 |
| CD8So | 14/04/2016 | H26 | *Pleuronectes platessa* | 45 | 987 |
| CD9So | 14/04/2016 | H26 | *Pleuronectes platessa* | 27.5 | 184 |
| CD13St | 14/04/2016 | H25 | *Asterias rubens* | 5.59 | 9.3 |
| CD14St | 14/04/2016 | H25 | *Asterias rubens* | 7.58 | 11.43 |
| CD17St | 14/04/2016 | H25 | *Asterias rubens* | 6.4 | 9.45 |
| CD18St | 14/04/2016 | H25 | *Asterias rubens* | 5.98 | 6.11 |
| CD22St | 14/04/2016 | H25 | *Asterias rubens* | 5.2 | 5.32 |
| CD17K | 14/04/2016 | H25 | *Limanda limanda* | 21 | 112 |
| CD12F | 14/04/2016 | H25 | *Platichthys flesus* | 27 | 196 |
| CD13F | 14/04/2016 | H25 | *Platichthys flesus* | 23.5 | 136 |
| CD37H | 14/04/2016 | H24 | *Clupea harengus* | 19 | 50 |
| CD39H | 14/04/2016 | H24 | *Clupea harengus* | 22 | 72 |
| CD42H | 14/04/2016 | H24 | *Clupea harengus* | 12 | 11.47 |
| CD45H | 14/04/2016 | H24 | *Clupea harengus* | 25 | 108 |
| CD46H | 14/04/2016 | H24 | *Clupea harengus* | 22 | 78 |
| CD182S | 14/04/2016 | H24 | *Sprattus sprattus* | 13 | 16.13 |
| CD183S | 14/04/2016 | H24 | *Sprattus sprattus* | 11.5 | 11.95 |
| CD184S | 14/04/2016 | H24 | *Sprattus sprattus* | 13 | 16.06 |
| CD187S | 14/04/2016 | H24 | *Sprattus sprattus* | 13 | 15.38 |
| CD63S | 14/04/2016 | H24 | *Sprattus sprattus* | 12 | 12.62 |
| CD14F | 15/04/2016 | H19 | *Platichthys flesus* | 37 | 397 |
| CD15F | 15/04/2016 | H19 | *Platichthys flesus* | 25.5 | 176 |
| CD15W | 15/04/2016 | H19 | *Merlangius merlangus* | 35 | 405 |
| CD20W | 15/04/2016 | H19 | *Merlangius merlangus* | 29 | 221 |
| CD21W | 15/04/2016 | H19 | *Merlangius merlangus* | 32 | 224 |
| CD29W | 15/04/2016 | H19 | *Merlangius merlangus* | 30 | 202 |
| CD30W | 15/04/2016 | H19 | *Merlangius merlangus* | 31 | 256 |
| CD50H | 16/04/2016 | BB29 | *Clupea harengus* | 17 | 26 |
| CD52H | 16/04/2016 | BB29 | *Clupea harengus* | 17 | 38 |
| CD53H | 16/04/2016 | BB29 | *Clupea harengus* | 17 | 30 |
| CD57H | 16/04/2016 | BB29 | *Clupea harengus* | 16.5 | 36 |
| CD58H | 16/04/2016 | BB29 | *Clupea harengus* | 15.5 | 36 |
| CD105S | 16/04/2016 | BB29 | *Sprattus sprattus* | 11 | 8.18 |
| CD75S | 16/04/2016 | BB29 | *Sprattus sprattus* | 12.5 | 12.34 |
| CD84S | 16/04/2016 | BB29 | *Sprattus sprattus* | 11 | 9.06 |
| CD87S | 16/04/2016 | BB29 | *Sprattus sprattus* | 11.5 | 12.23 |
| CD91S | 16/04/2016 | BB29 | *Sprattus sprattus* | 12.5 | 11.99 |
| CD109S | 17/04/2016 | GD57 | *Sprattus sprattus* | 9.5 | 6.48 |
| CD110S | 17/04/2016 | GD57 | *Sprattus sprattus* | 10.5 | 7.27 |
| CD112S | 17/04/2016 | GD57 | *Sprattus sprattus* | 10.5 | 6.91 |
| CD114S | 17/04/2016 | GD57 | *Sprattus sprattus* | 11 | 8.28 |
| CD115S | 17/04/2016 | GD57 | *Sprattus sprattus* | 10.5 | 7.02 |
| CD60H | 17/04/2016 | GD57 | *Clupea harengus* | 20 | 46 |
| CD61H | 17/04/2016 | GD57 | *Clupea harengus* | 22.5 | 66 |
| CD63H | 17/04/2016 | GD57 | *Clupea harengus* | 17 | 22 |
| CD68H | 17/04/2016 | GD57 | *Clupea harengus* | 19 | 48 |
| CD69H | 17/04/2016 | GD57 | *Clupea harengus* | 21.5 | 56 |

Table S2. Amino acid δ^13^C values (mean±standard deviation; n=3).

| **ID** | **Ala** |  |  | **Asx** |  |  | **Glx** |  |  | **Gly** |  |  | **His** |  |  | **Ile** |  |  | **Leu** |  |  | **Lys** |  |  | **Met** |  |  | **Phe** |  |  | **Pro** |  |  | **Ser** |  |  | **Thr** |  |  | **Tyr** |  |  | **Val** |  |  |
| --- | --- | --- | --- | --- | --- | --- | --- | --- | --- | --- | --- | --- | --- | --- | --- | --- | --- | --- | --- | --- | --- | --- | --- | --- | --- | --- | --- | --- | --- | --- | --- | --- | --- | --- | --- | --- | --- | --- | --- | --- | --- | --- | --- | --- | --- |
| CD1K | -16.8 | ± | 0.1 | -17.6 | ± | 0.2 | -17.1 | ± | 0.2 | -12.2 | ± | 0.4 | -18.1 | ± | 0.3 | -22.5 | ± | 0.8 | -27.5 | ± | 0.5 | -17.1 | ± | 0.3 | -23.5 | ± | 0.9 | -28.5 | ± | 0.3 | -18.8 | ± | 0.3 | -4.0 | ± | 0.3 | -6.4 | ± | 0.3 | -26.0 | ± | 0.3 | -23.5 | ± | 0.4 |
| CD3K | -16.7 | ± | 0.3 | -18.3 | ± | 0.1 | -17.4 | ± | 0.1 | -12.7 | ± | 0.0 | -17.8 | ± | 0.6 | -22.6 | ± | 0.4 | -28.1 | ± | 0.7 | -16.4 | ± | 0.2 | -23.5 | ± | 0.4 | -28.5 | ± | 0.1 | -19.2 | ± | 0.4 | -4.1 | ± | 0.4 | -6.9 | ± | 0.1 | -25.8 | ± | 0.3 | -24.3 | ± | 0.2 |
| CD4K | -16.0 | ± | 0.3 | -19.2 | ± | 0.1 | -18.3 | ± | 0.2 | -8.8 | ± | 0.3 | -16.7 | ± | 0.2 | -22.9 | ± | 0.2 | -27.9 | ± | 0.4 | -17.0 | ± | 0.0 | -23.9 | ± | 0.3 | -29.2 | ± | 0.3 | -18.4 | ± | 0.1 | 1.9 | ± | 0.3 | -7.0 | ± | 0.2 | -26.6 | ± | 0.5 | -24.2 | ± | 0.3 |
| CD6K | -17.0 | ± | 0.2 | -18.4 | ± | 0.3 | -18.2 | ± | 0.1 | -10.8 | ± | 0.1 | -17.7 | ± | 0.2 | -22.4 | ± | 0.5 | -28.0 | ± | 0.3 | -16.3 | ± | 0.2 | -22.8 | ± | 0.4 | -28.4 | ± | 0.0 | -18.6 | ± | 0.4 | -2.5 | ± | 0.5 | -7.1 | ± | 0.3 | -26.1 | ± | 0.2 | -23.7 | ± | 0.5 |
| CD8K | -16.7 | ± | 0.3 | -18.7 | ± | 0.2 | -17.9 | ± | 0.1 | -9.2 | ± | 0.0 | -17.5 | ± | 0.2 | -22.3 | ± | 0.7 | -28.0 | ± | 0.5 | -16.1 | ± | 0.2 | -22.3 | ± | 0.6 | -28.6 | ± | 0.1 | -18.3 | ± | 0.4 | -2.4 | ± | 0.3 | -7.3 | ± | 0.2 | -25.9 | ± | 0.3 | -23.9 | ± | 0.1 |
| CD1F | -16.2 | ± | 0.3 | -17.9 | ± | 0.1 | -16.6 | ± | 0.2 | -8.1 | ± | 0.1 | -17.1 | ± | 0.2 | -21.5 | ± | 0.5 | -27.0 | ± | 0.5 | -15.9 | ± | 0.1 | -21.4 | ± | 0.4 | -27.9 | ± | 0.2 | -18.0 | ± | 0.2 | -2.1 | ± | 0.4 | -6.4 | ± | 0.4 | -25.7 | ± | 0.3 | -23.3 | ± | 0.2 |
| CD2F | -17.7 | ± | 0.2 | -20.0 | ± | 0.1 | -18.7 | ± | 0.2 | -5.9 | ± | 0.3 | -17.2 | ± | 0.1 | -22.9 | ± | 0.4 | -28.9 | ± | 0.3 | -16.9 | ± | 0.0 | -22.9 | ± | 0.3 | -29.0 | ± | 0.1 | -19.7 | ± | 0.1 | -1.7 | ± | 0.4 | -9.1 | ± | 0.3 | -26.7 | ± | 0.4 | -24.9 | ± | 0.2 |
| CD1M | -20.7 | ± | 0.6 | -18.8 | ± | 0.1 | -19.3 | ± | 0.2 | -12.2 | ± | 0.2 | -20.2 | ± | 0.3 | -22.6 | ± | 0.9 | -29.6 | ± | 0.3 | -16.5 | ± | 0.2 | -22.2 | ± | 0.1 | -29.2 | ± | 0.2 | -21.6 | ± | 0.2 | -11.2 | ± | 0.5 | -10.2 | ± | 0.2 | -27.3 | ± | 0.1 | -26.5 | ± | 0.2 |
| CD3M | -20.3 | ± | 0.4 | -18.4 | ± | 0.0 | -18.2 | ± | 0.1 | -13.4 | ± | 0.2 | -20.3 | ± | 0.4 | -21.5 | ± | 0.0 | -29.2 | ± | 0.5 | -15.1 | ± | 0.2 | -20.5 | ± | 0.6 | -29.0 | ± | 0.1 | -20.9 | ± | 0.2 | -11.0 | ± | 0.5 | -8.7 | ± | 0.5 | -26.3 | ± | 0.2 | -25.0 | ± | 0.1 |
| CD4M | -20.0 | ± | 0.3 | -18.2 | ± | 0.1 | -17.9 | ± | 0.0 | -12.3 | ± | 0.5 | -20.2 | ± | 0.2 | -21.5 | ± | 0.2 | -28.7 | ± | 0.4 | -15.4 | ± | 0.2 | -20.7 | ± | 0.7 | -28.1 | ± | 0.1 | -21.4 | ± | 0.3 | -11.3 | ± | 0.4 | -8.6 | ± | 0.3 | -26.6 | ± | 0.0 | -25.2 | ± | 0.1 |
| CD5M | -21.0 | ± | 0.5 | -18.6 | ± | 0.1 | -18.8 | ± | 0.2 | -13.1 | ± | 0.1 | -17.8 | ± | 0.4 | -22.4 | ± | 0.5 | -29.2 | ± | 0.4 | -16.1 | ± | 0.0 | -21.8 | ± | 0.4 | -28.6 | ± | 0.2 | -21.5 | ± | 0.2 | -12.0 | ± | 0.5 | -10.2 | ± | 0.1 | -26.9 | ± | 0.0 | -25.9 | ± | 0.2 |
| CD6M | -20.0 | ± | 0.6 | -18.4 | ± | 0.2 | -18.9 | ± | 0.2 | -11.2 | ± | 0.3 | -20.0 | ± | 0.2 | -21.4 | ± | 0.2 | -29.0 | ± | 0.2 | -15.3 | ± | 0.2 | -20.7 | ± | 0.2 | -28.3 | ± | 0.2 | -20.7 | ± | 0.1 | -12.3 | ± | 0.4 | -9.5 | ± | 0.3 | -26.5 | ± | 0.1 | -25.5 | ± | 0.2 |
| CD1St | -14.6 | ± | 0.8 | -16.5 | ± | 0.2 | -15.1 | ± | 0.6 | 1.1 | ± | 0.4 | -16.1 | ± | 0.1 | -20.6 | ± | 0.6 | -27.8 | ± | 0.1 | -14.6 | ± | 0.2 | -22.1 | ± | 0.4 | -27.5 | ± | 0.4 | -19.5 | ± | 0.4 | 3.6 | ± | 0.7 | -3.8 | ± | 0.6 | -25.0 | ± | 0.2 | -22.3 | ± | 0.3 |
| CD2St | -17.2 | ± | 0.5 | -17.9 | ± | 0.3 | -17.1 | ± | 0.3 | -5.5 | ± | 0.3 | -16.6 | ± | 0.4 | -22.1 | ± | 0.4 | -28.4 | ± | 0.4 | -16.3 | ± | 0.2 | -23.5 | ± | 0.4 | -28.4 | ± | 0.3 | -20.3 | ± | 0.5 | -1.4 | ± | 0.2 | -5.8 | ± | 0.6 | -26.2 | ± | 0.2 | -23.4 | ± | 0.4 |
| CD4St | -16.0 | ± | 0.6 | -17.1 | ± | 0.1 | -15.9 | ± | 0.5 | -1.7 | ± | 0.1 | -15.9 | ± | 0.8 | -21.8 | ± | 0.8 | -28.1 | ± | 0.3 | -16.0 | ± | 0.4 | -23.0 | ± | 0.5 | -27.9 | ± | 0.4 | -19.8 | ± | 0.7 | 2.5 | ± | 0.7 | -4.3 | ± | 0.1 | -25.4 | ± | 0.2 | -22.7 | ± | 0.4 |
| CD7St | -17.0 | ± | 0.7 | -16.8 | ± | 0.6 | -16.2 | ± | 0.5 | -4.7 | ± | 0.0 | -17.2 | ± | 0.4 | -21.8 | ± | 0.7 | -28.3 | ± | 0.2 | -16.7 | ± | 1.0 | -23.7 | ± | 0.5 | -27.9 | ± | 0.3 | -20.0 | ± | 0.6 | -0.5 | ± | 0.4 | -4.6 | ± | 0.5 | -25.5 | ± | 0.1 | -23.3 | ± | 0.4 |
| CD9St | -15.6 | ± | 1.0 | -16.5 | ± | 0.1 | -15.6 | ± | 0.7 | -1.8 | ± | 0.2 | -16.3 | ± | 0.3 | -21.7 | ± | 0.5 | -28.1 | ± | 0.3 | -15.9 | ± | 0.4 | -22.2 | ± | 0.4 | -27.5 | ± | 0.4 | -19.5 | ± | 0.5 | 0.0 | ± | 0.6 | -4.5 | ± | 0.4 | -25.0 | ± | 0.4 | -23.0 | ± | 0.1 |
| CD1Sn | -17.4 | ± | 0.4 | -17.9 | ± | 0.2 | -18.0 | ± | 0.2 | -11.1 | ± | 0.0 | -16.9 | ± | 0.4 | -20.9 | ± | 0.2 | -28.3 | ± | 0.2 | -16.0 | ± | 0.2 | -23.4 | ± | 0.7 | -27.0 | ± | 0.2 | -20.0 | ± | 0.1 | -3.2 | ± | 0.6 | -8.0 | ± | 0.3 | -26.7 | ± | 0.1 | -23.8 | ± | 0.3 |
| CD2Sn | -16.5 | ± | 0.4 | -17.6 | ± | 0.1 | -18.1 | ± | 0.1 | -5.7 | ± | 0.2 | -17.0 | ± | 0.2 | -20.2 | ± | 0.2 | -27.6 | ± | 0.4 | -14.5 | ± | 0.1 | -22.8 | ± | 0.4 | -26.8 | ± | 0.2 | -18.9 | ± | 0.2 | -0.5 | ± | 0.7 | -6.7 | ± | 0.3 | -24.7 | ± | 0.1 | -23.3 | ± | 0.6 |
| CD3Sn | -16.6 | ± | 0.3 | -19.0 | ± | 0.1 | -19.8 | ± | 0.1 | -5.1 | ± | 0.2 | -18.3 | ± | 0.3 | -20.1 | ± | 0.2 | -28.3 | ± | 0.1 | -15.2 | ± | 0.1 | -22.2 | ± | 0.1 | -27.1 | ± | 0.2 | -19.3 | ± | 0.1 | -0.8 | ± | 0.7 | -8.0 | ± | 0.2 | -25.7 | ± | 0.1 | -23.7 | ± | 0.2 |
| CD5F | -15.7 | ± | 0.3 | -17.9 | ± | 0.1 | -15.7 | ± | 0.3 | -6.6 | ± | 0.2 | -16.3 | ± | 0.1 | -20.8 | ± | 0.2 | -26.3 | ± | 0.4 | -14.5 | ± | 0.1 | -21.5 | ± | 0.4 | -27.5 | ± | 0.3 | -17.6 | ± | 0.0 | -0.6 | ± | 0.3 | -6.6 | ± | 0.3 | -24.5 | ± | 0.4 | -22.8 | ± | 0.2 |
| CD6F | -16.9 | ± | 0.3 | -19.9 | ± | 0.3 | -18.0 | ± | 0.4 | -9.5 | ± | 0.1 | -17.5 | ± | 0.1 | -22.1 | ± | 0.2 | -27.8 | ± | 0.6 | -16.1 | ± | 0.2 | -22.8 | ± | 0.1 | -28.1 | ± | 0.5 | -19.4 | ± | 0.1 | -3.2 | ± | 0.0 | -8.1 | ± | 0.2 | -25.9 | ± | 0.2 | -23.8 | ± | 0.1 |
| CD8F | -8.3 | ± | 0.2 | -12.5 | ± | 0.1 | -10.3 | ± | 0.1 | -3.8 | ± | 0.1 | -13.4 | ± | 0.3 | -15.4 | ± | 0.6 | -21.2 | ± | 0.6 | -9.1 | ± | 0.2 | -15.9 | ± | 0.8 | -21.7 | ± | 0.0 | -12.5 | ± | 0.2 | 2.1 | ± | 0.5 | -1.9 | ± | 0.3 | -19.1 | ± | 0.3 | -17.0 | ± | 0.1 |
| CD14H | -19.0 | ± | 0.1 | -19.0 | ± | 0.1 | -17.5 | ± | 0.5 | -14.0 | ± | 0.4 | -19.9 | ± | 0.6 | -24.2 | ± | 0.9 | -28.8 | ± | 0.5 | -18.5 | ± | 0.3 | -23.9 | ± | 0.7 | -30.4 | ± | 0.3 | -19.4 | ± | 0.6 | -9.1 | ± | 0.4 | -8.6 | ± | 0.6 | -28.0 | ± | 0.1 | -24.7 | ± | 0.3 |
| CD1H | -18.6 | ± | 0.2 | -18.5 | ± | 0.1 | -17.4 | ± | 0.3 | -12.9 | ± | 0.1 | -18.6 | ± | 0.9 | -23.7 | ± | 0.4 | -28.6 | ± | 0.8 | -17.7 | ± | 0.3 | -23.5 | ± | 0.7 | -28.9 | ± | 0.2 | -17.6 | ± | 0.3 | -6.7 | ± | 0.4 | -7.8 | ± | 0.2 | -27.3 | ± | 0.4 | -24.3 | ± | 0.2 |
| CD5H | -19.0 | ± | 0.2 | -20.0 | ± | 0.2 | -18.2 | ± | 0.1 | -14.9 | ± | 0.1 | -20.2 | ± | 0.3 | -24.5 | ± | 0.3 | -28.6 | ± | 0.5 | -19.0 | ± | 0.2 | -24.3 | ± | 0.7 | -30.4 | ± | 0.0 | -19.8 | ± | 0.4 | -8.9 | ± | 0.3 | -8.1 | ± | 0.2 | -28.3 | ± | 0.3 | -25.4 | ± | 0.1 |
| CD7H | -18.2 | ± | 0.1 | -18.5 | ± | 0.0 | -16.6 | ± | 0.4 | -13.0 | ± | 0.3 | -19.8 | ± | 0.6 | -22.6 | ± | 0.6 | -28.2 | ± | 0.8 | -18.1 | ± | 0.3 | -23.0 | ± | 1.0 | -29.9 | ± | 0.2 | -19.0 | ± | 0.4 | -7.6 | ± | 0.4 | -6.7 | ± | 0.4 | -27.8 | ± | 0.3 | -22.8 | ± | 0.2 |
| CD8H | -19.2 | ± | 0.4 | -18.9 | ± | 0.2 | -17.1 | ± | 0.3 | -15.4 | ± | 0.3 | -19.8 | ± | 0.4 | -23.6 | ± | 0.3 | -28.5 | ± | 0.6 | -17.7 | ± | 0.2 | -24.3 | ± | 0.7 | -29.3 | ± | 0.2 | -19.0 | ± | 0.5 | -9.3 | ± | 0.6 | -7.7 | ± | 0.3 | -27.4 | ± | 0.1 | -24.3 | ± | 0.1 |
| CD163S | -17.3 | ± | 0.3 | -17.7 | ± | 0.1 | -16.0 | ± | 0.3 | -12.6 | ± | 0.1 | -19.9 | ± | 0.2 | -21.8 | ± | 0.4 | -26.5 | ± | 0.4 | -15.8 | ± | 0.1 | -22.7 | ± | 0.4 | -27.5 | ± | 0.2 | -17.4 | ± | 0.3 | -7.1 | ± | 0.4 | -5.7 | ± | 0.2 | -25.4 | ± | 0.1 | -23.2 | ± | 0.0 |
| CD168S | -19.0 | ± | 0.6 | -19.2 | ± | 0.1 | -17.2 | ± | 0.4 | -14.1 | ± | 0.1 | -19.7 | ± | 0.4 | -23.2 | ± | 0.1 | -28.3 | ± | 0.2 | -17.5 | ± | 0.2 | -24.1 | ± | 0.4 | -29.3 | ± | 0.4 | -18.5 | ± | 0.3 | -9.5 | ± | 0.6 | -7.3 | ± | 0.2 | -27.0 | ± | 0.3 | -24.5 | ± | 0.2 |
| CD172S | -19.3 | ± | 0.4 | -19.2 | ± | 0.1 | -17.6 | ± | 0.3 | -13.3 | ± | 0.1 | -19.6 | ± | 0.1 | -23.0 | ± | 0.1 | -28.5 | ± | 0.3 | -18.0 | ± | 0.1 | -24.4 | ± | 0.5 | -29.6 | ± | 0.2 | -18.6 | ± | 0.3 | -10.1 | ± | 0.6 | -7.5 | ± | 0.3 | -27.3 | ± | 0.1 | -24.5 | ± | 0.1 |
| CD174S | -18.6 | ± | 0.5 | -18.7 | ± | 0.1 | -17.3 | ± | 0.3 | -13.6 | ± | 0.1 | -19.8 | ± | 0.2 | -22.5 | ± | 0.3 | -27.8 | ± | 0.1 | -17.2 | ± | 0.2 | -23.6 | ± | 0.5 | -28.9 | ± | 0.1 | -17.9 | ± | 0.2 | -8.7 | ± | 0.6 | -6.5 | ± | 0.2 | -26.8 | ± | 0.2 | -24.2 | ± | 0.1 |
| CD175S | -18.8 | ± | 0.5 | -19.0 | ± | 0.1 | -17.9 | ± | 0.3 | -13.8 | ± | 0.3 | -19.9 | ± | 0.2 | -23.0 | ± | 0.2 | -28.5 | ± | 0.3 | -17.6 | ± | 0.2 | -24.0 | ± | 0.4 | -29.0 | ± | 0.3 | -19.0 | ± | 0.3 | -9.8 | ± | 0.5 | -7.8 | ± | 0.2 | -26.5 | ± | 0.2 | -24.8 | ± | 0.2 |
| CD11M | -19.3 | ± | 0.1 | -18.5 | ± | 0.0 | -18.7 | ± | 0.2 | -14.6 | ± | 0.1 | -21.0 | ± | 0.3 | -23.1 | ± | 0.5 | -27.6 | ± | 0.3 | -16.5 | ± | 0.1 | -21.0 | ± | 0.6 | -28.5 | ± | 0.1 | -20.8 | ± | 0.1 | -9.2 | ± | 0.2 | -8.1 | ± | 0.4 | -27.3 | ± | 0.4 | -25.3 | ± | 0.2 |
| CD13M | -19.9 | ± | 0.2 | -18.8 | ± | 0.2 | -18.8 | ± | 0.2 | -13.7 | ± | 0.2 | -20.0 | ± | 0.4 | -23.9 | ± | 0.1 | -29.7 | ± | 0.5 | -17.3 | ± | 0.1 | -21.5 | ± | 0.5 | -29.2 | ± | 0.2 | -20.3 | ± | 0.2 | -9.9 | ± | 0.2 | -10.3 | ± | 0.4 | -28.0 | ± | 0.3 | -26.8 | ± | 0.1 |
| CD14M | -19.4 | ± | 0.1 | -18.8 | ± | 0.1 | -19.2 | ± | 0.3 | -14.1 | ± | 0.1 | -20.5 | ± | 0.5 | -23.4 | ± | 0.3 | -29.7 | ± | 0.5 | -17.2 | ± | 0.2 | -21.1 | ± | 0.7 | -28.9 | ± | 0.2 | -20.9 | ± | 0.2 | -10.4 | ± | 0.3 | -9.6 | ± | 0.3 | -27.2 | ± | 0.4 | -26.5 | ± | 0.2 |
| CD7M | -20.3 | ± | 0.2 | -19.3 | ± | 0.2 | -19.1 | ± | 0.3 | -15.4 | ± | 0.2 | -20.5 | ± | 0.1 | -23.9 | ± | 0.4 | -28.5 | ± | 0.4 | -17.0 | ± | 0.1 | -21.8 | ± | 0.5 | -29.3 | ± | 0.2 | -21.0 | ± | 0.2 | -11.0 | ± | 0.2 | -9.8 | ± | 0.5 | -28.2 | ± | 0.3 | -27.0 | ± | 0.4 |
| CD8M | -20.0 | ± | 0.3 | -18.9 | ± | 0.1 | -19.1 | ± | 0.2 | -13.7 | ± | 0.1 | -19.1 | ± | 0.1 | -23.6 | ± | 0.2 | -29.1 | ± | 0.7 | -16.7 | ± | 0.2 | -21.2 | ± | 0.5 | -28.8 | ± | 0.2 | -20.6 | ± | 0.2 | -10.6 | ± | 0.2 | -9.6 | ± | 0.3 | -27.4 | ± | 0.3 | -26.4 | ± | 0.2 |
| CD11F | -16.8 | ± | 0.1 | -19.0 | ± | 0.1 | -17.9 | ± | 0.1 | -9.3 | ± | 0.2 | -17.5 | ± | 0.2 | -22.2 | ± | 0.8 | -27.3 | ± | 0.4 | -16.3 | ± | 0.2 | -22.8 | ± | 0.3 | -28.8 | ± | 0.2 | -18.6 | ± | 0.3 | -3.5 | ± | 0.4 | -8.2 | ± | 0.2 | -26.2 | ± | 0.4 | -23.2 | ± | 0.4 |
| CD10So | -17.7 | ± | 0.3 | -19.3 | ± | 0.5 | -19.7 | ± | 0.2 | -7.7 | ± | 0.5 | -18.5 | ± | 0.3 | -23.7 | ± | 0.7 | -29.3 | ± | 0.4 | -18.3 | ± | 0.1 | -23.6 | ± | 0.5 | -30.2 | ± | 0.2 | -20.1 | ± | 0.6 | -3.0 | ± | 0.3 | -9.2 | ± | 0.6 | -27.8 | ± | 0.4 | -25.2 | ± | 0.6 |
| CD11So | -17.5 | ± | 0.1 | -20.1 | ± | 0.4 | -19.7 | ± | 0.2 | -10.1 | ± | 0.4 | -18.2 | ± | 0.1 | -23.6 | ± | 0.7 | -29.0 | ± | 0.3 | -17.5 | ± | 0.1 | -22.8 | ± | 0.6 | -29.6 | ± | 0.1 | -20.8 | ± | 0.7 | -1.7 | ± | 0.5 | -9.0 | ± | 0.6 | -27.2 | ± | 0.3 | -25.2 | ± | 0.6 |
| CD12So | -18.4 | ± | 0.4 | -20.5 | ± | 0.2 | -19.4 | ± | 0.2 | -10.9 | ± | 0.1 | -19.1 | ± | 0.2 | -23.3 | ± | 0.4 | -28.2 | ± | 0.2 | -17.3 | ± | 0.1 | -23.6 | ± | 0.2 | -29.8 | ± | 0.2 | -20.2 | ± | 0.3 | -1.8 | ± | 0.5 | -9.0 | ± | 0.6 | -27.5 | ± | 0.3 | -25.6 | ± | 0.2 |
| CD8So | -17.8 | ± | 0.3 | -19.4 | ± | 0.1 | -18.5 | ± | 0.2 | -7.2 | ± | 0.2 | -18.9 | ± | 0.3 | -23.0 | ± | 0.2 | -28.6 | ± | 0.1 | -16.5 | ± | 0.2 | -22.6 | ± | 0.5 | -29.4 | ± | 0.4 | -20.2 | ± | 0.2 | -1.1 | ± | 0.3 | -8.0 | ± | 0.4 | -27.2 | ± | 0.5 | -25.6 | ± | 0.1 |
| CD9So | -17.5 | ± | 0.3 | -19.6 | ± | 0.4 | -19.6 | ± | 0.2 | -9.3 | ± | 0.4 | -18.9 | ± | 0.1 | -23.8 | ± | 0.5 | -29.0 | ± | 0.4 | -17.9 | ± | 0.3 | -23.5 | ± | 0.5 | -30.0 | ± | 0.0 | -20.2 | ± | 0.5 | -2.2 | ± | 0.3 | -8.9 | ± | 0.6 | -27.5 | ± | 0.3 | -25.4 | ± | 0.6 |
| CD13St | -15.3 | ± | 0.6 | -17.6 | ± | 0.4 | -16.8 | ± | 0.3 | -0.6 | ± | 0.1 | -20.6 | ± | 0.6 | -22.1 | ± | 0.5 | -29.2 | ± | 0.3 | -16.5 | ± | 0.2 | -23.4 | ± | 0.9 | -28.4 | ± | 0.3 | -18.6 | ± | 0.3 | 0.0 | ± | 0.4 | -7.2 | ± | 0.5 | -25.3 | ± | 0.6 | -23.5 | ± | 0.4 |
| CD14St | -15.7 | ± | 0.4 | -18.3 | ± | 0.2 | -17.0 | ± | 0.1 | -1.8 | ± | 0.4 | -15.6 | ± | 0.3 | -22.3 | ± | 0.4 | -29.4 | ± | 0.4 | -16.7 | ± | 0.3 | -23.5 | ± | 0.4 | -28.5 | ± | 0.1 | -19.3 | ± | 0.2 | 0.4 | ± | 0.5 | -7.6 | ± | 0.2 | -26.3 | ± | 0.2 | -24.0 | ± | 0.3 |
| CD17St | -15.7 | ± | 0.3 | -18.2 | ± | 0.2 | -17.5 | ± | 0.2 | -0.7 | ± | 0.1 | -16.2 | ± | 0.3 | -22.0 | ± | 0.3 | -29.3 | ± | 0.3 | -16.5 | ± | 0.0 | -23.0 | ± | 0.7 | -28.5 | ± | 0.2 | -19.8 | ± | 0.3 | -0.4 | ± | 0.4 | -7.3 | ± | 0.3 | -26.1 | ± | 0.4 | -24.0 | ± | 0.5 |
| CD18St | -16.5 | ± | 0.5 | -17.8 | ± | 0.4 | -16.5 | ± | 0.3 | -3.3 | ± | 0.1 | -15.2 | ± | 0.1 | -21.9 | ± | 0.3 | -28.4 | ± | 0.3 | -16.1 | ± | 0.1 | -23.7 | ± | 0.7 | -27.8 | ± | 0.4 | -19.1 | ± | 0.3 | -1.1 | ± | 0.3 | -6.9 | ± | 0.5 | -26.3 | ± | 0.1 | -22.8 | ± | 0.7 |
| CD22St | -14.2 | ± | 0.7 | -15.9 | ± | 0.5 | -15.5 | ± | 0.4 | 2.6 | ± | 0.3 | -15.4 | ± | 0.4 | -21.8 | ± | 0.5 | -28.2 | ± | 0.3 | -16.3 | ± | 0.2 | -23.5 | ± | 0.7 | -28.0 | ± | 0.3 | -17.6 | ± | 0.1 | 1.9 | ± | 0.2 | -5.8 | ± | 0.2 | -26.3 | ± | 0.1 | -23.0 | ± | 0.3 |
| CD17K | -16.1 | ± | 0.3 | -16.0 | ± | 0.2 | -15.6 | ± | 0.2 | -9.3 | ± | 0.3 |  |  |  | -23.3 | ± | 0.9 | -28.2 | ± | 0.3 | -15.7 | ± | 0.3 | -25.2 | ± | 0.0 | -28.9 | ± | 0.1 | -18.9 | ± | 0.9 | -5.1 | ± | 0.4 | -9.6 | ± | 0.3 | -26.7 | ± | 0.2 | -25.5 | ± | 0.6 |
| CD12F | -18.3 | ± | 0.1 | -20.2 | ± | 0.3 | -20.5 | ± | 0.1 | -4.6 | ± | 0.1 | -18.5 | ± | 0.3 | -24.2 | ± | 0.9 | -29.8 | ± | 0.3 | -18.6 | ± | 0.2 | -23.5 | ± | 0.2 | -30.7 | ± | 0.3 | -20.3 | ± | 0.7 | -1.9 | ± | 0.2 | -9.7 | ± | 0.4 | -28.3 | ± | 0.6 | -26.1 | ± | 0.7 |
| CD13F | -18.0 | ± | 0.2 | -20.4 | ± | 0.1 | -19.7 | ± | 0.1 | -9.7 | ± | 0.1 | -19.2 | ± | 0.3 | -24.0 | ± | 0.9 | -29.5 | ± | 0.2 | -18.1 | ± | 0.2 | -23.3 | ± | 0.4 | -30.1 | ± | 0.1 | -20.1 | ± | 0.5 | -4.5 | ± | 0.5 | -9.5 | ± | 0.2 | -28.1 | ± | 0.2 | -25.3 | ± | 0.7 |
| CD37H | -18.4 | ± | 0.3 | -19.3 | ± | 0.2 | -17.6 | ± | 0.1 | -12.7 | ± | 0.1 | -18.7 | ± | 0.7 | -22.2 | ± | 0.1 | -27.9 | ± | 0.4 | -17.4 | ± | 0.1 | -23.0 | ± | 0.6 | -29.3 | ± | 0.3 | -17.8 | ± | 0.3 | -8.0 | ± | 0.5 | -6.5 | ± | 0.1 | -27.6 | ± | 0.2 | -23.5 | ± | 0.1 |
| CD39H | -17.9 | ± | 0.1 | -19.3 | ± | 0.2 | -17.9 | ± | 0.2 | -12.7 | ± | 0.0 | -18.3 | ± | 0.4 | -22.2 | ± | 0.2 | -27.9 | ± | 0.3 | -16.6 | ± | 0.1 | -22.4 | ± | 0.6 | -29.0 | ± | 0.3 | -17.4 | ± | 0.2 | -7.1 | ± | 0.4 | -6.3 | ± | 0.1 | -27.0 | ± | 0.3 | -23.5 | ± | 0.1 |
| CD42H | -20.0 | ± | 0.2 | -20.7 | ± | 0.3 | -18.7 | ± | 0.1 | -14.9 | ± | 0.3 | -20.4 | ± | 0.2 | -24.6 | ± | 0.2 | -29.1 | ± | 0.3 | -19.3 | ± | 0.1 | -25.1 | ± | 0.6 | -30.8 | ± | 0.3 | -19.6 | ± | 0.2 | -9.8 | ± | 0.3 | -9.4 | ± | 0.1 | -28.9 | ± | 0.2 | -25.5 | ± | 0.1 |
| CD45H | -17.7 | ± | 0.2 | -17.2 | ± | 0.0 | -16.1 | ± | 0.3 | -11.4 | ± | 0.2 | -18.9 | ± | 0.2 | -21.1 | ± | 0.2 | -27.6 | ± | 0.8 | -16.0 | ± | 0.2 | -21.3 | ± | 0.6 | -27.9 | ± | 0.3 | -16.6 | ± | 0.2 | -6.8 | ± | 0.4 | -5.5 | ± | 0.2 | -26.1 | ± | 0.3 | -23.6 | ± | 0.4 |
| CD46H | -18.2 | ± | 0.2 | -18.1 | ± | 0.3 | -17.2 | ± | 0.1 | -13.0 | ± | 0.2 | -19.0 | ± | 0.4 | -22.1 | ± | 0.1 | -27.2 | ± | 0.5 | -16.8 | ± | 0.2 | -22.2 | ± | 0.8 | -28.5 | ± | 0.0 | -17.7 | ± | 0.4 | -7.3 | ± | 0.4 | -6.2 | ± | 0.1 | -27.0 | ± | 0.4 | -23.8 | ± | 0.2 |
| CD182S | -17.7 | ± | 0.1 | -19.4 | ± | 0.2 | -17.8 | ± | 0.1 | -13.2 | ± | 0.4 | -18.6 | ± | 0.3 | -24.0 | ± | 0.6 | -28.8 | ± | 0.1 | -18.2 | ± | 0.2 | -23.8 | ± | 0.7 | -29.7 | ± | 0.1 | -18.9 | ± | 0.5 | -7.0 | ± | 0.4 | -9.2 | ± | 0.4 | -27.5 | ± | 0.4 | -24.7 | ± | 0.6 |
| CD183S | -19.7 | ± | 0.2 | -19.4 | ± | 0.1 | -17.4 | ± | 0.2 | -14.3 | ± | 0.2 | -20.0 | ± | 0.3 | -23.8 | ± | 0.2 | -28.7 | ± | 0.2 | -18.9 | ± | 0.3 | -24.9 | ± | 0.6 | -30.0 | ± | 0.3 | -19.1 | ± | 0.2 | -10.3 | ± | 0.5 | -8.2 | ± | 0.0 | -28.2 | ± | 0.3 | -25.1 | ± | 0.2 |
| CD184S | -18.1 | ± | 0.2 | -19.9 | ± | 0.1 | -18.4 | ± | 0.2 | -13.4 | ± | 0.5 | -19.5 | ± | 0.1 | -23.1 | ± | 0.5 | -29.2 | ± | 0.3 | -18.7 | ± | 0.2 | -22.9 | ± | 1.0 | -30.7 | ± | 0.6 | -19.8 | ± | 0.5 | -7.5 | ± | 0.5 | -7.8 | ± | 0.4 | -27.7 | ± | 0.4 | -23.5 | ± | 0.4 |
| CD187S | -18.7 | ± | 0.2 | -20.3 | ± | 0.3 | -18.6 | ± | 0.5 | -12.6 | ± | 0.5 | -19.3 | ± | 0.4 | -25.3 | ± | 1.1 | -29.7 | ± | 0.1 | -19.8 | ± | 0.4 | -24.8 | ± | 0.3 | -30.9 | ± | 0.1 | -19.5 | ± | 0.5 | -8.1 | ± | 0.6 | -9.4 | ± | 0.6 | -28.2 | ± | 0.3 | -25.7 | ± | 0.5 |
| CD63S | -19.6 | ± | 0.2 | -19.1 | ± | 0.1 | -17.5 | ± | 0.1 | -13.3 | ± | 0.1 | -20.2 | ± | 0.4 | -24.5 | ± | 0.5 | -29.1 | ± | 0.5 | -19.0 | ± | 0.1 | -25.2 | ± | 0.5 | -30.6 | ± | 0.1 | -19.1 | ± | 0.3 | -9.3 | ± | 0.5 | -8.5 | ± | 0.1 | -28.3 | ± | 0.2 | -25.2 | ± | 0.1 |
| CD14F | -17.2 | ± | 0.2 | -19.6 | ± | 0.3 | -18.2 | ± | 0.1 | -10.0 | ± | 0.2 | -18.7 | ± | 0.4 | -22.8 | ± | 0.7 | -28.2 | ± | 0.1 | -17.2 | ± | 0.3 | -22.9 | ± | 0.5 | -29.0 | ± | 0.3 | -19.4 | ± | 0.6 | -5.1 | ± | 0.5 | -7.5 | ± | 0.4 | -27.0 | ± | 0.4 | -24.3 | ± | 0.6 |
| CD15F | -18.1 | ± | 0.2 | -19.9 | ± | 0.3 | -19.1 | ± | 0.2 | -9.4 | ± | 0.2 | -19.8 | ± | 0.5 | -23.7 | ± | 1.0 | -29.1 | ± | 0.5 | -18.1 | ± | 0.5 | -23.0 | ± | 0.9 | -30.1 | ± | 0.2 | -19.8 | ± | 0.1 | -4.7 | ± | 0.3 | -9.2 | ± | 0.1 | -27.9 | ± | 0.3 | -25.3 | ± | 0.5 |
| CD15W | -17.8 | ± | 0.0 | -22.3 | ± | 0.3 | -19.6 | ± | 0.3 | -8.0 | ± | 0.3 | -18.3 | ± | 0.2 | -24.0 | ± | 0.3 | -29.0 | ± | 0.2 | -18.8 | ± | 0.2 | -24.9 | ± | 0.4 | -31.6 | ± | 0.4 | -18.3 | ± | 0.5 | -2.4 | ± | 0.4 | -8.8 | ± | 0.5 | -27.8 | ± | 0.4 | -24.4 | ± | 0.6 |
| CD20W | -16.3 | ± | 0.2 | -21.0 | ± | 0.1 | -18.2 | ± | 0.1 | -9.2 | ± | 0.3 | -17.3 | ± | 0.2 | -23.6 | ± | 0.5 | -28.4 | ± | 0.4 | -17.8 | ± | 0.1 | -24.9 | ± | 0.2 | -31.4 | ± | 0.4 | -17.8 | ± | 0.2 | -1.6 | ± | 0.2 | -8.2 | ± | 0.2 | -27.1 | ± | 0.3 | -24.6 | ± | 0.3 |
| CD21W | -16.9 | ± | 0.1 | -20.7 | ± | 0.2 | -17.6 | ± | 0.2 | -9.7 | ± | 0.5 | -18.1 | ± | 0.5 | -23.1 | ± | 0.4 | -27.6 | ± | 0.2 | -17.8 | ± | 0.3 | -25.2 | ± | 0.5 | -30.8 | ± | 0.2 | -17.4 | ± | 0.2 | -1.5 | ± | 0.4 | -7.2 | ± | 0.3 | -27.3 | ± | 0.4 | -23.7 | ± | 0.6 |
| CD29W | -16.3 | ± | 0.2 | -19.7 | ± | 0.3 | -17.0 | ± | 0.2 | -9.8 | ± | 0.9 | -16.8 | ± | 0.4 | -23.7 | ± | 0.8 | -27.9 | ± | 0.3 | -17.7 | ± | 0.3 | -25.3 | ± | 0.5 | -30.5 | ± | 0.2 | -17.3 | ± | 0.3 | -0.5 | ± | 0.4 | -7.4 | ± | 0.3 | -27.4 | ± | 0.3 | -23.6 | ± | 0.5 |
| CD30W | -17.9 | ± | 0.1 | -21.0 | ± | 0.3 | -18.1 | ± | 0.3 | -9.2 | ± | 0.5 | -17.4 | ± | 0.1 | -23.7 | ± | 0.8 | -28.2 | ± | 0.1 | -18.0 | ± | 0.4 | -24.3 | ± | 0.8 | -31.0 | ± | 0.3 | -18.3 | ± | 0.4 | -2.6 | ± | 0.5 | -7.2 | ± | 0.6 | -27.4 | ± | 0.4 | -23.9 | ± | 0.6 |
| CD50H | -18.2 | ± | 0.2 | -18.7 | ± | 0.3 | -17.8 | ± | 0.0 | -11.5 | ± | 0.4 | -19.0 | ± | 0.3 | -23.9 | ± | 0.5 | -28.7 | ± | 0.1 | -18.4 | ± | 0.2 | -23.2 | ± | 0.7 | -30.4 | ± | 0.4 | -17.8 | ± | 0.4 | -6.3 | ± | 0.3 | -9.6 | ± | 0.3 | -27.4 | ± | 0.2 | -24.9 | ± | 0.7 |
| CD52H | -18.0 | ± | 0.1 | -18.5 | ± | 0.2 | -17.4 | ± | 0.2 | -10.4 | ± | 0.1 | -18.5 | ± | 0.6 | -23.2 | ± | 0.4 | -28.2 | ± | 0.2 | -16.9 | ± | 0.0 | -23.6 | ± | 0.5 | -29.2 | ± | 0.1 | -16.8 | ± | 0.4 | -7.4 | ± | 0.6 | -8.1 | ± | 0.0 | -27.0 | ± | 0.4 | -24.0 | ± | 0.2 |
| CD53H | -18.0 | ± | 0.2 | -18.1 | ± | 0.1 | -17.1 | ± | 0.4 | -10.5 | ± | 0.4 | -18.5 | ± | 0.5 | -23.7 | ± | 0.7 | -27.9 | ± | 0.4 | -18.2 | ± | 0.4 | -22.9 | ± | 0.7 | -29.4 | ± | 0.3 | -17.4 | ± | 0.5 | -5.7 | ± | 0.6 | -8.6 | ± | 0.4 | -27.1 | ± | 0.3 | -24.4 | ± | 0.7 |
| CD57H | -18.9 | ± | 0.1 | -19.4 | ± | 0.1 | -17.7 | ± | 0.2 | -13.0 | ± | 0.2 | -19.9 | ± | 0.3 | -24.2 | ± | 0.2 | -28.6 | ± | 0.2 | -18.4 | ± | 0.0 | -25.1 | ± | 0.4 | -30.1 | ± | 0.2 | -17.8 | ± | 0.2 | -8.7 | ± | 0.7 | -7.9 | ± | 0.1 | -28.6 | ± | 0.3 | -24.6 | ± | 0.1 |
| CD58H | -19.5 | ± | 0.1 | -21.1 | ± | 0.2 | -18.9 | ± | 0.2 | -15.1 | ± | 0.2 | -19.9 | ± | 0.5 | -24.0 | ± | 0.2 | -28.2 | ± | 0.2 | -18.5 | ± | 0.1 | -24.3 | ± | 0.2 | -30.4 | ± | 0.2 | -18.7 | ± | 0.2 | -8.7 | ± | 0.7 | -8.3 | ± | 0.3 | -28.2 | ± | 0.3 | -24.8 | ± | 0.2 |
| CD105S | -18.9 | ± | 0.3 | -17.8 | ± | 0.2 | -16.4 | ± | 0.4 | -13.5 | ± | 0.2 | -18.6 | ± | 0.3 | -23.4 | ± | 0.1 | -27.6 | ± | 0.4 | -17.8 | ± | 0.5 | -23.8 | ± | 0.5 | -29.5 | ± | 0.2 | -17.6 | ± | 0.4 | -9.3 | ± | 0.4 | -7.3 | ± | 0.4 | -26.9 | ± | 0.2 | -24.8 | ± | 0.3 |
| CD75S | -18.9 | ± | 0.2 | -19.0 | ± | 0.2 | -17.5 | ± | 0.2 | -13.4 | ± | 0.3 | -20.0 | ± | 0.6 | -23.9 | ± | 0.3 | -28.7 | ± | 0.6 | -18.9 | ± | 0.3 | -24.2 | ± | 0.8 | -30.1 | ± | 0.1 | -18.6 | ± | 0.3 | -10.5 | ± | 0.4 | -8.2 | ± | 0.1 | -27.9 | ± | 0.2 | -24.7 | ± | 0.2 |
| CD84S | -19.1 | ± | 0.2 | -19.5 | ± | 0.2 | -18.3 | ± | 0.2 | -13.3 | ± | 0.4 | -19.9 | ± | 0.6 | -24.8 | ± | 0.5 | -29.4 | ± | 0.6 | -18.9 | ± | 0.3 | -24.1 | ± | 0.8 | -30.5 | ± | 0.1 | -18.9 | ± | 0.5 | -10.0 | ± | 0.4 | -9.0 | ± | 0.0 | -28.0 | ± | 0.2 | -25.2 | ± | 0.1 |
| CD87S | -19.1 | ± | 0.2 | -19.2 | ± | 0.2 | -18.2 | ± | 0.1 | -14.0 | ± | 0.3 | -19.7 | ± | 0.3 | -24.3 | ± | 0.5 | -29.2 | ± | 0.6 | -19.1 | ± | 0.2 | -24.5 | ± | 0.7 | -30.3 | ± | 0.1 | -19.0 | ± | 0.4 | -9.6 | ± | 0.4 | -8.9 | ± | 0.1 | -27.9 | ± | 0.2 | -25.3 | ± | 0.1 |
| CD91S | -19.2 | ± | 0.2 | -19.3 | ± | 0.1 | -17.5 | ± | 0.1 | -12.8 | ± | 0.0 | -19.0 | ± | 0.1 | -23.2 | ± | 0.2 | -27.9 | ± | 0.5 | -17.2 | ± | 0.1 | -23.3 | ± | 0.9 | -29.3 | ± | 0.2 | -18.3 | ± | 0.2 | -7.8 | ± | 0.4 | -6.9 | ± | 0.1 | -27.0 | ± | 0.2 | -23.6 | ± | 0.1 |
| CD109S | -19.4 | ± | 0.2 | -20.7 | ± | 0.1 | -18.0 | ± | 0.3 | -14.4 | ± | 0.3 | -18.9 | ± | 0.1 | -23.9 | ± | 0.2 | -28.9 | ± | 0.5 | -18.6 | ± | 0.4 | -23.5 | ± | 0.4 | -29.9 | ± | 0.1 | -18.5 | ± | 0.4 | -8.4 | ± | 0.3 | -7.9 | ± | 0.4 | -27.3 | ± | 0.2 | -25.4 | ± | 0.4 |
| CD110S | -19.8 | ± | 0.2 | -20.0 | ± | 0.2 | -18.3 | ± | 0.4 | -14.8 | ± | 0.2 | -19.3 | ± | 0.6 | -24.6 | ± | 0.0 | -29.8 | ± | 0.6 | -19.3 | ± | 0.4 | -24.9 | ± | 0.5 | -31.1 | ± | 0.3 | -19.2 | ± | 0.6 | -9.9 | ± | 0.5 | -8.3 | ± | 0.6 | -28.1 | ± | 0.2 | -26.3 | ± | 0.4 |
| CD112S | -19.1 | ± | 0.3 | -18.6 | ± | 0.3 | -16.4 | ± | 0.5 | -13.0 | ± | 0.2 | -18.7 | ± | 0.1 | -23.6 | ± | 0.1 | -28.7 | ± | 0.6 | -18.5 | ± | 0.5 | -23.9 | ± | 0.7 | -29.9 | ± | 0.3 | -18.1 | ± | 0.5 | -9.6 | ± | 0.5 | -7.9 | ± | 0.4 | -27.1 | ± | 0.3 | -25.4 | ± | 0.4 |
| CD114S | -19.7 | ± | 0.3 | -19.3 | ± | 0.3 | -17.8 | ± | 0.5 | -12.7 | ± | 0.2 | -18.9 | ± | 0.3 | -24.1 | ± | 0.1 | -29.5 | ± | 0.5 | -18.9 | ± | 0.5 | -24.4 | ± | 0.4 | -30.5 | ± | 0.3 | -18.7 | ± | 0.4 | -8.8 | ± | 0.4 | -9.1 | ± | 0.4 | -27.4 | ± | 0.2 | -26.3 | ± | 0.3 |
| CD115S | -19.1 | ± | 0.2 | -17.8 | ± | 0.2 | -16.1 | ± | 0.2 | -14.1 | ± | 0.2 | -18.6 | ± | 0.6 | -23.3 | ± | 0.1 | -27.8 | ± | 0.2 | -17.8 | ± | 0.4 | -23.9 | ± | 0.7 | -29.4 | ± | 0.1 | -17.9 | ± | 0.4 | -9.0 | ± | 0.3 | -7.3 | ± | 0.2 | -26.7 | ± | 0.1 | -25.0 | ± | 0.2 |
| CD60H | -18.1 | ± | 0.5 | -19.0 | ± | 0.1 | -16.8 | ± | 0.1 | -11.7 | ± | 0.4 | -18.0 | ± | 0.1 | -23.1 | ± | 0.0 | -28.4 | ± | 0.8 | -17.3 | ± | 0.1 | -22.9 | ± | 0.6 | -28.9 | ± | 0.2 | -17.5 | ± | 0.2 | -6.2 | ± | 0.3 | -8.3 | ± | 0.4 | -26.9 | ± | 0.1 | -24.6 | ± | 0.0 |
| CD61H | -18.7 | ± | 0.4 | -19.4 | ± | 0.1 | -18.2 | ± | 0.2 | -11.2 | ± | 0.0 | -17.8 | ± | 0.3 | -23.9 | ± | 0.5 | -29.1 | ± | 0.3 | -18.2 | ± | 0.1 | -23.4 | ± | 0.5 | -29.4 | ± | 0.2 | -17.8 | ± | 0.5 | -6.9 | ± | 0.2 | -10.2 | ± | 0.2 | -27.1 | ± | 0.1 | -24.7 | ± | 0.5 |
| CD63H | -18.6 | ± | 0.4 | -18.6 | ± | 0.2 | -17.9 | ± | 0.1 | -12.7 | ± | 0.3 | -19.2 | ± | 0.5 | -23.7 | ± | 0.6 | -27.8 | ± | 0.6 | -17.8 | ± | 0.1 | -22.9 | ± | 0.7 | -29.5 | ± | 0.1 | -17.6 | ± | 0.4 | -6.2 | ± | 0.2 | -8.7 | ± | 0.2 | -27.1 | ± | 0.3 | -24.7 | ± | 0.4 |
| CD68H | -19.2 | ± | 0.4 | -20.2 | ± | 0.3 | -18.8 | ± | 0.2 | -13.3 | ± | 0.2 | -17.6 | ± | 0.4 | -23.9 | ± | 0.4 | -28.6 | ± | 0.4 | -18.4 | ± | 0.0 | -23.7 | ± | 0.4 | -29.3 | ± | 0.1 | -18.2 | ± | 0.2 | -5.8 | ± | 0.4 | -9.6 | ± | 0.4 | -27.5 | ± | 0.1 | -24.9 | ± | 0.1 |
| CD69H | -18.1 | ± | 0.3 | -18.8 | ± | 0.3 | -18.3 | ± | 0.3 | -10.4 | ± | 0.4 | -17.8 | ± | 0.4 | -23.6 | ± | 0.4 | -27.7 | ± | 0.3 | -17.7 | ± | 0.3 | -23.0 | ± | 0.2 | -29.2 | ± | 0.3 | -17.4 | ± | 0.5 | -5.9 | ± | 0.3 | -9.8 | ± | 0.4 | -26.8 | ± | 0.1 | -24.5 | ± | 0.7 |

Table S3. Non-lipid corrected (nlc) and lipid-corrected (lc; according to Kiljunen *et al.* 2006*) bulk δ^13^C and δ^15^N values, and carbon to nitrogen ratios.

| ID | δ^13^C_nlc_ (‰; VPDB) | δ^13^C_lc_ (‰; VPDB) | δ^15^N_nlc_ (‰; AIR) | C:N |  | ID | δ^13^C (‰; VPDB) | δ^13^C_lc_ (‰; VPDB) | δ^15^N_nlc_ (‰; AIR) | C:N |
| --- | --- | --- | --- | --- | --- | --- | --- | --- | --- | --- |
| CD10So | -20.9 | -18.1 | 11.6 | 4.96 |  | CD22St | -20.2 | -17.2 | 9.8 | 5.20 |
| CD11F | -20.3 | -18.6 | 15.0 | 3.95 |  | CD2F | -20.1 | -18.1 | 12.6 | 4.15 |
| CD11M | -21.8 | -19.2 | 6.7 | 4.70 |  | CD2St | -19.7 | -17.0 | 10.8 | 4.81 |
| CD11So | -21.3 | -18.5 | 11.2 | 4.88 |  | CD37H | -21.9 | -20.0 | 12.8 | 4.09 |
| CD12F | -21.8 | -19.6 | 9.9 | 4.35 |  | CD39H | -21.4 | -19.7 | 12.6 | 3.93 |
| CD12So | -21.1 | -18.1 | 11.5 | 5.13 |  | CD3K | -20.5 | -18.8 | 12.4 | 3.98 |
| CD13F | -21.7 | -19.9 | 12.1 | 4.08 |  | CD3M | -20.2 | -17.4 | 10.0 | 4.94 |
| CD13M | -22.1 | -19.4 | 7.8 | 4.73 |  | CD42H | -22.8 | -21.0 | 11.5 | 3.96 |
| CD13St | -21.5 | -18.0 | 8.5 | 5.82 |  | CD45H | -21.6 | -18.8 | 12.9 | 4.95 |
| CD14F | -21.2 | -19.4 | 11.2 | 4.01 |  | CD46H | -21.3 | -19.3 | 13.0 | 4.18 |
| CD14H | -22.4 | -20.2 | 12.6 | 4.36 |  | CD4K | -21.0 | -19.0 | 12.0 | 4.15 |
| CD14M | -22.0 | -19.5 | 6.6 | 4.59 |  | CD4M | -19.9 | -17.2 | 9.6 | 4.80 |
| CD14St | -20.2 | -17.0 | 10.3 | 5.48 |  | CD4St | -19.3 | -16.5 | 12.6 | 4.95 |
| CD15F | -21.8 | -19.7 | 11.1 | 4.24 |  | CD5F | -18.2 | -16.4 | 11.5 | 4.01 |
| CD163S | -21.4 | -19.4 | 11.7 | 4.18 |  | CD5H | -23.9 | -21.3 | 11.5 | 4.67 |
| CD168S | -23.4 | -20.9 | 11.1 | 4.62 |  | CD5M | -20.0 | -17.3 | 10.0 | 4.77 |
| CD16K | -22.0 | -19.1 | 12.9 | 5.06 |  | CD63S | -25.2 | -21.5 | 11.0 | 6.18 |
| CD172S | -23.3 | -20.5 | 11.6 | 4.92 |  | CD6F | -19.6 | -17.6 | 11.8 | 4.16 |
| CD174S | -22.9 | -20.2 | 12.5 | 4.81 |  | CD6K | -20.5 | -18.7 | 13.1 | 4.02 |
| CD175S | -22.0 | -20.2 | 13.0 | 4.01 |  | CD6M | -20.0 | -17.3 | 9.6 | 4.78 |
| CD17K | -20.8 | -19.0 | 12.1 | 4.01 |  | CD7H | -22.5 | -20.4 | 12.7 | 4.25 |
| CD17St | -21.2 | -17.7 | 9.5 | 5.88 |  | CD7M | -21.9 | -19.7 | 7.8 | 4.40 |
| CD183S | -24.3 | -21.2 | 10.6 | 5.25 |  | CD7St | -20.0 | -16.7 | 11.2 | 5.65 |
| CD18St | -20.4 | -17.5 | 9.6 | 5.08 |  | CD8H | -23.3 | -20.3 | 11.6 | 5.06 |
| CD1F | -19.7 | -17.8 | 13.4 | 4.11 |  | CD8K | -20.2 | -18.2 | 13.2 | 4.18 |
| CD1H | -21.6 | -19.8 | 11.7 | 4.00 |  | CD8M | -20.9 | -18.5 | 6.9 | 4.54 |
| CD1K | -20.6 | -18.8 | 12.1 | 4.00 |  | CD8So | -20.8 | -18.1 | 11.3 | 4.76 |
| CD1M | -20.6 | -18.0 | 10.1 | 4.69 |  | CD9So | -21.4 | -18.7 | 11.1 | 4.76 |
| CD1St | -19.3 | -16.1 | 10.9 | 5.45 |  |  |  |  |  |  |

* Kiljunen, M., Grey, J., Sinisalo, T., Harrod, C., Immonen, H. & Jones, R.I. (2006) A revised model for lipid-normalizing δ^13^C values from aquatic organisms, with implications for isotope mixing models. **43**, 1213-1222.

**Supplementary Figure**

Figure S1. Linear discriminant function analysis based on a combined δ^13^C_EAA_ dataset of herring and sprat. The two first linear discriminants shown here explains 54.1% (LD1) and 24.6% (LD2) of the variability – the remaining linear discriminant LD3 explains 21.3%. For LD1, the two greatest coefficients are tyrosine (2.3) and isoleucine (-0.7), for LD2 it is phenylalanine (-2.2) and lysine (2.0), and for LD3 it is isoleucine (1.8) and lysine (-1.1). Despite the relatively large overlap in the three LD scores among the four sites, the site group median values are significantly different (Pillai’s Trace = 0.82, F_9,108_ = 4.5; P < 0.001).


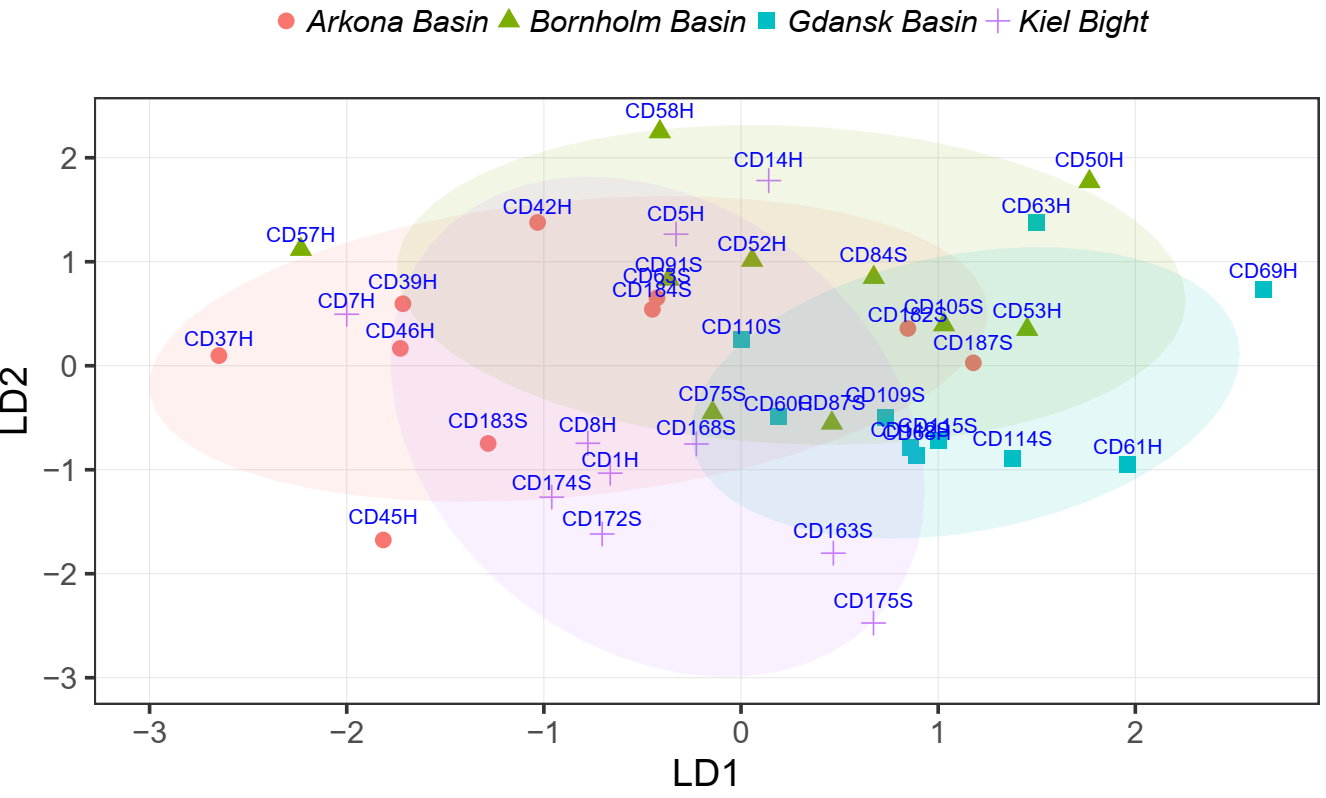

Supplement: Supplementary file 1 — Supplementary Material [file ECE3-10-7768-s001.docx]
